# Association between health insurance cost-sharing and choice of hospital tier for cardiovascular diseases in China: a prospective cohort study

**DOI:** 10.1016/j.lanwpc.2024.101020

**Published:** 2024-02-14

**Authors:** Muriel Levy, John Buckell, Robert Clarke, Nina Wu, Pei Pei, Dianjianyi Sun, Daniel Avery, Hua Zhang, Jun Lv, Canqing Yu, Liming Li, Zhengming Chen, Winnie Yip, Yiping Chen, Borislava Mihaylova, China Kadoorie Biobank (CKB) Collaborative Group, Junshi Chen, Zhengming Chen, Robert Clarke, Rory Collins, Liming Li, Chen Wang, Jun Lv, Richard Peto, Robin Walters, Daniel Avery, Maxim Barnard, Derrick Bennett, Ruth Boxall, Kahung Chan, Yiping Chen, Zhengming Chen, Johnathan Clarke, Robert Clarke, Huaidong Du, Ahmed Edris Mohamed, Hannah Fry, Simon Gilbert, Pek Kei Im, Andri Iona, Maria Kakkoura, Christiana Kartsonaki, Hubert Lam, Kuang Lin, James Liu, Mohsen Mazidi, Iona Millwood, Sam Morris, Qunhua Nie, Alfred Pozaricki, Paul Ryder, Saredo Said, Dan Schmidt, Becky Stevens, Iain Turnbull, Robin Walters, Baihan Wang, Lin Wang, Neil Wright, Ling Yang, Xiaoming Yang, Pang Yao, Xiao Han, Can Hou, Qingmei Xia, Chao Liu, Jun Lv, Pei Pei, Dianjianyi Sun, Canqing Yu, Naying Chen, Duo Liu, Zhenzhu Tang, Ningyu Chen, Qilian Jiang, Jian Lan, Mingqiang Li, Yun Liu, Fanwen Meng, Jinhuai Meng, Rong Pan, Yulu Qin, Ping Wang, Sisi Wang, Liuping Wei, Liyuan Zhou, Caixia Dong, Pengfei Ge, Xiaolan Ren, Zhongxiao Li, Enke Mao, Tao Wang, Hui Zhang, Xi Zhang, Jinyan Chen, Ximin Hu, Xiaohuan Wang, Zhendong Guo, Huimei Li, Yilei Li, Min Weng, Shukuan Wu, Shichun Yan, Mingyuan Zou, Xue Zhou, Ziyan Guo, Quan Kang, Yanjie Li, Bo Yu, Qinai Xu, Liang Chang, Lei Fan, Shixian Feng, Ding Zhang, Gang Zhou, Yulian Gao, Tianyou He, Pan He, Chen Hu, Huarong Sun, Xukui Zhang, Biyun Chen, Zhongxi Fu, Yuelong Huang, Huilin Liu, Qiaohua Xu, Li Yin, Huajun Long, Xin Xu, Hao Zhang, Libo Zhang, Jian Su, Ran Tao, Ming Wu, Jie Yang, Jinyi Zhou, Yonglin Zhou, Yihe Hu, Yujie Hua, Jianrong Jin, Fang Liu, Jingchao Liu, Yan Lu, Liangcai Ma, Aiyu Tang, Jun Zhang, Liang Cheng, Ranran Du, Ruqin Gao, Feifei Li, Shanpeng Li, Yongmei Liu, Feng Ning, Zengchang Pang, Xiaohui Sun, Xiaocao Tian, Shaojie Wang, Yaoming Zhai, Hua Zhang, Wei Hou, Silu Lv, Junzheng Wang, Xiaofang Chen, Xianping Wu, Ningmei Zhang, Xiaoyu Chang, Xiaofang Chen, Jianguo Li, Jiaqiu Liu, Guojin Luo, Qiang Sun, Xunfu Zhong, Weiwei Gong, Ruying Hu, Hao Wang, Meng Wang, Min Yu, Lingli Chen, Qijun Gu, Dongxia Pan, Chunmei Wang, Kaixu Xie, Xiaoyi Zhang, Hongyuan Chen, Liyang Liu, Haiyan Gou, Xun Wang, Jing Ding, Ning Zhang, Yueshi Mao, Shanshan Zhou, Lirong Jin, Xin Cheng, Yun Lu, Li Chen, Zilong Hao, Xiaona Xing, Lei Wang, Naixin Ju, Yiting Mao, Shuya Li, Peng Du, Deren Wang, Xiaojia Sun, Shihao You, Weizhi Wang, Yanmei Zhu, Xiaojiu Li, Yi Dong

**Affiliations:** a Clinical Trial Service Unit and Epidemiological Studies Unit (CTSU), Nuffield Department of Population Health, University of Oxford, UK; b Health Economics Research Centre, Nuffield Department of Population Health, University of Oxford, UK; c Medical Research Council Population Health Research Unit (MRC PHRU), Nuffield Department of Population Health, University of Oxford, UK; d School of Public Health, Capital Medical University, Beijing, China; e Peking University Center for Public Health and Epidemic Preparedness & Response, Beijing, China; f Department of Epidemiology and Biostatistics, School of Public Health, Peking University Health Science Centre, Beijing, China; g NCDs Prevention and Control Department, Qingdao Centre for Disease Control and Prevention, Qingdao, China; h Harvard T H Chan School of Public Health, Boston, MA, USA; i Health Economics and Policy Research Unit, Wolfson Institute of Population Health, Queen Mary University of London, London, UK

**Keywords:** Hospital type, Cardiovascular diseases, Healthcare seeking behaviour, China

## Abstract

**Background:**

Hospitals in China are classified into tiers (1, 2 or 3), with the largest (tier 3) having more equipment and specialist staff. Differential health insurance cost-sharing by hospital tier (lower deductibles and higher reimbursement rates in lower tiers) was introduced to reduce overcrowding in higher tier hospitals, promote use of lower tier hospitals, and limit escalating healthcare costs. However, little is known about the effects of differential cost-sharing in health insurance schemes on choice of hospital tiers.

**Methods:**

In a 9-year follow-up of a prospective study of 0.5 M adults from 10 areas in China, we examined the associations between differential health insurance cost-sharing and choice of hospital tiers for patients with a first hospitalisation for stroke or ischaemic heart disease (IHD) in 2009–2017. Analyses were performed separately in urban areas (stroke: n = 20,302; IHD: n = 19,283) and rural areas (stroke: n = 21,130; IHD: n = 17,890), using conditional logit models and adjusting for individual socioeconomic and health characteristics.

**Findings:**

About 64–68% of stroke and IHD cases in urban areas and 27–29% in rural areas chose tier 3 hospitals. In urban areas, higher reimbursement rates in each tier and lower tier 3 deductibles were associated with a greater likelihood of choosing their respective hospital tiers. In rural areas, the effects of cost-sharing were modest, suggesting a greater contribution of other factors. Higher socioeconomic status and greater disease severity were associated with a greater likelihood of seeking care in higher tier hospitals in urban and rural areas.

**Interpretation:**

Patient choice of hospital tiers for treatment of stroke and IHD in China was influenced by differential cost-sharing in urban areas, but not in rural areas. Further strategies are required to incentivise appropriate health seeking behaviour and promote more efficient hospital use.

**Funding:**

10.13039/100010269Wellcome Trust, 10.13039/501100000265Medical Research Council, 10.13039/501100000274British Heart Foundation, 10.13039/501100000289Cancer Research UK, 10.13039/501100017647Kadoorie Charitable Foundation, China Ministry of Science and Technology, and 10.13039/501100001809National Natural Science Foundation of China.

**Research in context**

**Evidence before this study**

We searched for studies examining health insurance influence on choice of healthcare facilities in China, from January 1, 2004, to July, 13, 2023 using MEDLINE. The search strategy included the terms “health facilities” (or “health care provider”) and “choice” (or “health seeking”). Reference lists of identified relevant articles were also reviewed. We included studies based in mainland China and which had choice of healthcare facility level involving a hospital as the outcome of interest, and health insurance as a factor studied. Three studies examined the direct effects of reimbursement rates or deductibles on choice of healthcare facilities. One study reported that the deductible had an adverse effect and a higher reimbursement ceiling had a positive effect on health seeking behaviours in rural areas. Another study reported that increasing the ratio of average reimbursement rate for county or higher-level hospitals to the reimbursement rate for township healthcare centres increased the probability of choosing a higher-level healthcare facility for enrolees of the rural health insurance scheme. The third study reported that increasing both reimbursement rates and physician density at primary care facilities were associated with a greater likelihood of visiting primary care facilities and a lower probability of visiting county and higher-level hospitals.

**Added value of this study**

This is the first large study to analyse the choice of tier 1, 2 or 3 hospitals for inpatient care in urban and rural areas, using revealed preference data from health insurance claims rather than from surveys. The effect of cost sharing on choice of hospital tier differed between urban and rural areas. In urban areas, higher reimbursement rates in each tier and lower tier 3 deductibles were associated with a greater likelihood of choosing the respective hospital tiers. In rural areas, the influence of cost-sharing was limited, suggesting a greater contribution of other factors. In urban and rural areas, higher socioeconomic status, and greater disease severity were associated with a greater likelihood of choosing higher tier hospitals.

**Implications of all the available evidence**

The available evidence showed that the choice of hospital tier for treatment of common cardiovascular diseases was more sensitive to degree of cost-sharing among participants in urban than in rural areas in China. The differential impacts of socioeconomic characteristics on the choice of hospital tiers highlighted inequalities in access to different hospital tiers in China. These results could inform health insurance policies and healthcare reforms to promote the appropriate and efficient use of hospital tiers. They also indicate need to study the role of quality of care and workforce capacity in lower tier hospitals and of more efficient transfers or referrals to higher tier hospitals for more specialised care to optimise health seeking behaviours.

## Introduction

In the absence of a well-developed primary care and referral system in China,^1^ Chinese adults are free to choose to seek treatment at any hospital.^2^ Hospitals in China are classified into three tiers, with higher tier hospitals, typically located in urban areas, being larger and better equipped.^1^ The aim of the three-tier healthcare system was to provide more efficient healthcare in China, with tier 1 and 2 hospitals providing the basic medical care for the local population and tier 3 hospitals providing specialist care for major diseases. However, due to the lack of a well-developed primary care and referral system in China,^2^ patients can choose to visit a healthcare provider in any tier.^3^ Irrespective of the severity of their illness, patients prefer to seek medical treatment in higher rather than lower tier hospitals,^1^^,^^4^ leading to an overuse of tier 3 hospitals.^2^^,^^4^ The growing demand for hospital care in higher tier hospitals has resulted in substantial problems for delivery of healthcare, including increasing costs and inefficient allocation of resources, with tertiary hospitals accounting for the largest proportion of the overall health expenditure.

One of the chief objectives of the healthcare reforms launched in China in 2009 was to improve the primary care system and divert patient admissions from higher to lower tier hospitals.^5^^,^^6^ The Chinese government advocated changes to patient cost-sharing, establishment of medical alliances between large hospitals and primary care providers with a clear referral system, and an aspiration to register all patients with a family doctor.^7^ The use of differential health insurance (HI) cost-sharing by hospital tier (lower deductibles and higher reimbursement rates in lower tiers) has been adopted by some local governments to incentivise patients to seek treatment in lower tier hospitals, limit costs, and improve efficiency.

Until now, few studies have investigated the direct effects of HI cost-sharing and choice of hospital tiers in China. Brown and Theoharides (2009) demonstrated that conditional on seeking treatment and adjusting for demographic and socioeconomic factors and services covered by the HI scheme, the deductible had an adverse effect and a higher reimbursement ceiling had a positive effect on the choice of a given hospital type in rural areas.^8^ However, the effects of HI characteristics on choice of hospital tier were not studied. Hou et al. (2014) reported that a half unit increase in the ratio of average reimbursement rate for county or higher-level hospitals to the reimbursement rate for township healthcare centres increased the probability of choosing a higher-level healthcare facility by 0.34 for enrolees of the rural HI scheme.^9^ One nationally representative study reported that increasing both HI average reimbursement rates and physician density at primary care facilities was associated with a greater likelihood of visiting primary care facilities and a lower probability of visiting county and higher-level hospitals.^10^ When the average reimbursement rate at primary care facilities increased by 1 percentage point, the likelihood of inpatients choosing county-, municipal- and higher-level hospitals versus primary care facility decreased by 46%, 81% and 63%, respectively. Previous studies on the choice of healthcare providers in China typically focused on any healthcare, outpatient care and did not specify type of care,^11^, ^12^, ^13^, ^14^, ^15^, ^16^ and used self-reported data about provider choice from surveys.

We examined the association between differential health insurance cost-sharing and choice of hospital tiers for patients with stroke or ischaemic heart disease (IHD) in 2009–2017, using a 9-year follow-up of a prospective study of 0.5 M adults from 10 areas in China. In contrast with previous studies on choice of healthcare providers in China, this study focuses on inpatient hospital care for two major diseases, namely stroke and IHD, and uses individual participant data on provider choice from HI claims for the years 2009–2017. Cardiovascular diseases (CVD), chiefly stroke and IHD, were selected for the present analyses as these two diseases account for about 40% of all deaths in China.^17^ Diseases of the circulatory system also represented the largest share of city-wide hospital admissions among major disease categories in China in 2013–2017.^18^ The increasing number of patients with CVD has resulted in escalating financial pressures on the healthcare system and on individual patients and their families, with major inequalities between urban and rural areas.^19^

The study addresses the following hypotheses: (i) higher reimbursement rates in a given tier were associated with a greater likelihood of choosing such hospital tier, (ii) higher deductibles in a given tier were associated with a lower likelihood of choosing such tier, and (iii) the associations between cost-sharing and choice of hospital tiers differed between urban and rural areas in China. The study also took account of patient socioeconomic characteristics and other factors potentially influencing healthcare seeking behaviour.

## Methods

### Study design and participants

We used data from the China Kadoorie Biobank (CKB), a prospective cohort study of 0.5 million adults recruited from 10 areas (5 urban, 5 rural) in China between June 25, 2004, and July 15, 2008. The study period was restricted to hospital admissions after 2009, because recruitment to the CKB was completed on July 15, 2008, and comprehensive data from HI records were available from January 1, 2009. The urban study areas (i.e. cities) included Qingdao, Harbin, Haikou, Suzhou and Liuzhou, and the rural study areas (i.e. counties in the five provinces) included Sichuan, Gansu, Henan, Zhejiang and Hunan. Official local residential records were used to identify eligible individuals (adults aged 30–79 years). All participants completed an interviewer-administered questionnaire, providing information on demographic and socioeconomic characteristics, medical history, and lifestyle factors, and physical measurements. Hospital admissions data were obtained by linkage to HI system hospital records and to regional disease registers. Hospital admissions were identified using the International Classification of Diseases 10th revision (ICD-10) codes I60-I61 and I63-I64 for stroke, and I20-I25 for IHD. All first admission events of stroke and IHD were verified against retrieved medical records and adjudicated by specialists in CKB. All CKB participants alive on January 1, 2009 and admitted to any public or private hospital for inpatient care in the period 2009–2017 were included in the present study. Details of CKB study design, ethical approval and provision of informed consent for participation have been previously reported.^20^

### Dependent variables

The dependent variable was the choice of one of the three hospital tiers (Appendix Method A) for first admissions for stroke or IHD, respectively, among participants without a history of stroke, transient ischaemic attack or IHD. The study focused on first admissions with either stroke or IHD, since these were less likely to be influenced by previous healthcare experience than recurrent admissions.

### Independent variables involving health insurance characteristics

Details of characteristics of HI schemes available in each of the 10 study areas were collected using questionnaires completed by senior public health staff in each study area. The senior public health staff used official local policy documents and regulations from the social HI agencies to extract details for each year on reimbursement rates (% of eligible healthcare costs), deductibles (Yuan) per visit, and reimbursement ceiling (Yuan) for inpatient care for the years 2009–2017. Data for deductibles and reimbursement ceilings were standardised to 2017 values using urban/rural-specific consumer prices indices from the China statistical yearbooks.^21^ The HI characteristics varied by CKB centre, HI scheme, calendar year, and (except ceiling) hospital tier. In some CKB centres, reimbursement rates varied by level of hospitalisation costs and, for the urban employee HI scheme, by individual's retirement status. The changes in cost-sharing for inpatient care were directed by local governments, with the aim of incentivising the use of lower rather than higher tier hospitals. Consequently, there were natural exogenous variations in reimbursement rates, deductibles, and ceilings within and between CKB centres.

### Control variables including patient characteristics

The patient characteristics included demographic, socioeconomic, lifestyle and morbidity factors. Demographic factors included sex and age at admission. Socioeconomic factors included marital status, household size, highest level of education attained (no formal school, primary or middle school, high school or above) and annual household income in Yuan (<10,000, 10,000–19,999, 20,000–34,999, ≥35,000). Lifestyle factors included smoking and alcohol consumption, body mass index, physical activity, and self-rated health status. Socioeconomic and lifestyle factors were restricted to information collected at entry into CKB. In addition to stroke type (lacunar ischaemic stroke [IS], non-lacunar IS, and haemorrhagic stroke [HS]) and IHD type (acute myocardial infarction [AMI] or angina, and other IHD), morbidity factors included history of other major diseases, such as cancer, diabetes and diseases of the respiratory system. Except for body mass index, stroke or IHD type and morbidity factors, all other patient characteristics were self-reported by participants. The participant recruitment area and calendar year fixed effects were included to control for differences in unobserved characteristics in the 10 CKB areas, including health system characteristics, other HI differences, and secular time trends affecting all areas. Participants lived in either an urban or a rural CKB area, yet they could be enrolled in different HI schemes.

### Statistical analyses

Conditional logit (McFadden's) choice models were used to examine associations between HI cost-sharing and patient characteristics and choice of hospital tier (Appendices Methods B and C).^22^ This type of multinomial logit model was selected because the dependent variable, choice of hospital tier, a categorical variable (3 categories: tier 1, tier 2 or tier 3), could vary between patients and hospital tier characteristics. The parameters of the model were estimated using maximum-likelihood estimation. Tier-specific variables, namely reimbursement rates and deductibles, were specified for each hospital tier to obtain separate associations by tier. Case-specific variables, which did not vary by hospital tier, included demographic, socioeconomic, lifestyle and morbidity factors, reimbursement ceiling, and area and year-fixed effects. To assess possible heterogeneity in the effect of HI characteristics by socioeconomic status, the associations between HI characteristics and hospital tier choice were investigated by levels of income or education on admissions for both stroke and IHD. All analyses were performed separately in urban and rural areas, as the organisation and delivery of healthcare varies substantially between urban and rural areas in China.^3^

Forecasts of the impact of varying reimbursement rates or deductibles were made and own and cross-elasticities of choice were estimated using the sample enumeration technique.^23^ For such forecasts, the values of the reimbursement rates or deductibles were varied separately and together while holding all other variables constant, new probabilities were predicted for each individual and averaged over the sample. Confidence intervals were estimated using 10,000 runs of the prediction on sets of random draws of model coefficients, and then taking the 2.5th and 97.5th percentiles for the 95% confidence interval. Hausman tests and nested logit and mixed logit models did not indicate violations of the “independence of irrelevant alternatives” (IIA) assumption (Appendix Method C).^24^

### Sensitivity analyses

In sensitivity analyses, adjustments were limited to age, sex, year and region fixed effects only (minimally-adjusted models), to study the effect of adjusting for socioeconomic, lifestyle and morbidity factors on the coefficients for reimbursement rates and deductibles. Conditional logit models were also estimated: (i) with separate inclusion of either reimbursement rates or deductibles, (ii) without adjusting for ceiling, and (iii) using single coefficients for reimbursement rates and for deductibles across tiers (non-tier specific). Furthermore, models were estimated using calculated out-of-pocket (OOP) payments and actual reimbursement rates (ARR) instead of reimbursement rates and deductibles (Appendix Method D). Models were estimated with further adjustments for regional supply-side factors based on data from China statistical yearbooks, including the annual number of doctors (per 10,000 persons) and the number of beds by hospital tier (per 10,000). For comparison purposes, analyses were performed for first admissions for any cause. All analyses were conducted using Stata 16 or R 4.1.0.

### Ethical approval

Ethical approval for the CKB study was obtained from the Oxford Tropical Research Ethics Committee at the University of Oxford (approval number 025-04, 3rd February 2005) and the Chinese CDC Ethical Review Committee (approval number 005/2004, 9th July 2004). Approval was also granted by institutional research boards and the local CDCs in each of the survey areas.

### Role of the funding source

The funders had no role in the study design, data collection, data analysis and interpretation, writing of the report, or the decision to submit the article for publication.

## Results

### Study population characteristics

Among the 42,755 participants with first stroke admissions and 38,213 with first IHD admissions in the CKB between 2009 and 2017, 793 (1.9%) and 649 (1.7%) had missing data on hospital tiers and, independently, 583 (1.4%) and 417 (1.1%) had missing data on HI types or were uninsured, respectively. After excluding participants with missing data, 41,432 (urban: 20,302, rural: 21,130) participants with stroke and 37,173 (urban: 19,283, rural: 17,890) participants with IHD were included in the present analyses.

Among participants with stroke and IHD in urban and rural areas, the mean age at admission was 65–66 years and the majority of participants (55–62%) were women (Table 1). The proportions of participants who had completed high school education and those with an annual household income above ¥35,000 at entry were both greater among participants living in urban than in rural areas. In urban areas, between 82 and 88% of individuals were enrolled in the urban employee basic medical insurance (UEBMI), while in rural areas, approximately 90% of individuals were enrolled in the new rural cooperative medical scheme (NRCMS). The proportions of men who were current smokers were higher in rural than in urban areas, while the proportions of men who were regular alcohol drinkers were higher in urban than in rural areas. The mean levels of physical activity were higher in rural than in urban areas. Conversely, the proportions of individuals who were overweight or obese were higher in urban than in rural areas. The proportions of individuals who reported poor self-rated health were higher in rural than in urban areas.

**Table 1:** Selected characteristics of China Kadoorie Biobank participants with first stroke and ischaemic heart disease (IHD) admissions in urban and rural areas in 2009–2017.

	Stroke		IHD	
	Urban (n = 20,302)	Rural (n = 21,130)	Urban (n = 19,283)	Rural (n = 17,890)
**Demographic characteristics**				
Age (years) at admission, Mean (SD)	66.4 (9.8)	65.0 (9.7)	65.3 (9.9)	65.7 (10.0)
Women, %	56.6	54.5	62.1	59.6
**Socioeconomic characteristics**				
Married, %	85.3	87.3	86.1	86.5
Household size, Mean (SD)	3.1 (1.4)	4.2 (1.7)	3.0 (1.3)	4.1 (1.8)
Highest level of education attained, %				
No formal school	14.2	28.5	10.8	26.7
Primary/middle school	50.9	64.3	51.2	66.3
High school or above	34.9	7.2	38.1	7.1
Annual household income (¥), %				
<10,000	17.1	45.6	14.1	44.9
10,000–19,999	33.2	30.7	33.3	30.3
20,000–34,999	28.6	15.5	31.0	16.3
≥35,000	21.1	8.2	21.6	8.4
Health insurance type at admission, %				
URBMI or NRCMS^a^	18.4	91.4	12.0	89.4
UEBMI	81.6	8.6	88.0	10.6
**Lifestyle characteristics**				
Current smoker, %				
Women	4.2	3.5	3.9	4.6
Men	50.4	65.0	51.3	65.8
Regular alcohol drinker, %				
Women	4.9	3.6	5.9	3.9
Men	50.3	38.7	50.0	38.4
Physical activity (MET-h/day), Mean (SD)	14.1 (10.7)	19.0 (13.5)	13.8 (9.8)	18.0 (12.6)
Overweight or obese, %	46.6	33.7	50.7	34.0
**Morbidity factors, %**				
Poor self-rated health	9.8	14.0	10.1	17.2
Disease type at admission				
Haemorrhagic stroke	7.8	16.3		
Non-lacunar ischaemic stroke	71.3	76.7		
Lacunar ischaemic stroke	20.9	7.0		
AMI or angina			8.7	9.7
Other IHD			91.3	90.3

All characteristics were measured at baseline unless otherwise specified. AMI: acute myocardial infarction, IHD: ischaemic heart disease, MET: metabolic equivalents of task, NRCMS: new rural cooperative medical scheme, SD: standard deviation, UEBMI: urban employee basic medical insurance, URBMI: urban resident basic medical insurance.

a For presentation purposes, URBMI and NRCMS are presented together as they provided similar benefits and in 2012–2013 merged into a single scheme in four of the ten CKB areas. Generally, in urban areas, 5–10% of participants were enrolled in NRCMS and 7–8% of participants were enrolled in URBMI. In rural areas, 89–91% of participants were enrolled in NRCMS and 0.1% in URBMI.

### Health insurance characteristics

**Fig. 1:**
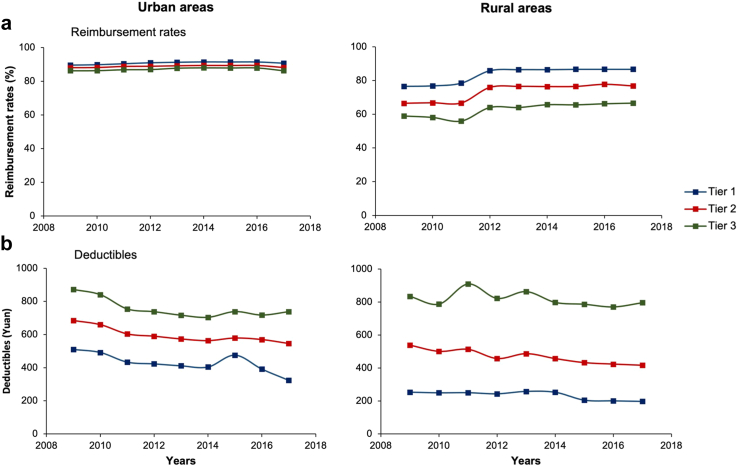
**Average reimbursement rates (a) and deductibles (b) by hospital tier in urban and rural CKB centres, between 2009 and 2017.** Data presented for China Kadoorie Biobank (CKB) participants with first stroke. Reimbursement rates and deductibles were averaged across individuals by HI scheme and region, within urban and rural areas. The averages were then weighted by the proportion of CKB participants with first stroke enrolled in each scheme in urban and rural areas. Deductibles were inflated to 2017 values.

Differential health insurance cost-sharing by hospital tier was adopted in all CKB areas between 2009 and 2017 (Appendix eTable 1). In urban CKB areas, although the average reimbursement rates remained stable over time and consistent between hospital tiers (Fig. 1), there was substantial variation between regions, calendar year and hospital tiers. The average levels of deductibles were highest in higher tier hospitals (Fig. 1). In 2009–2017, the average deductibles decreased by 4.9%, 2.8% and 2.0% per year in tier 1, 2 and 3 hospitals, respectively. While some urban centres had the same reimbursement rates and deductibles across tiers in 2009, an increasing number of CKB areas implemented differential cost-sharing by hospital tier by 2017. The average reimbursement ceiling increased over time from 174,712¥ in 2009 to 241,401¥ in 2017, or by approximately 4.2% per year. In rural CKB centres, the average reimbursement rates were highest in lower tier hospitals in 2009–2017, and increased by 1.6%, 1.9% and 1.7% per year in tier 1, tier 2 and tier 3 hospitals, respectively (Fig. 1). The average levels of deductibles were highest in higher tier hospitals and decreased by 2.8%, 3.0% and 0.3% per year in tier 1, tier 2 and tier 3 hospitals. On average, the reimbursement ceiling increased from 53,001¥ in 2009 to 133,607¥ in 2017 or by about 13% per year. The average reimbursement rates and ceilings remained higher in urban than in rural areas over time, and the average deductibles in tier 3 hospitals were higher in rural areas after 2010.

### Hospital admissions for stroke and IHD

In 2009–2017, the proportions of admissions for stroke in tier 1, tier 2 and tier 3 hospitals were: 6.0%, 30.4% and 63.5% in urban areas, and 29.9%, 40.7%, and 29.4% in rural areas, respectively. The corresponding proportions for IHD admissions were 5.9%, 25.8% 68.3% in urban areas, and 47.3%, 26.0% and 26.7% in rural areas. Between 2009 and 2017, in urban areas, the proportion of admissions in tier 1 and 2 hospitals increased for stroke (from 5.0% to 7.5% in tier 1 and from 28.2% to 31.8% in tier 2) and IHD (from 6.5% to 9.1% in tier 1 and from 22.7% to 26.4% in tier 2), while the proportion of admissions in tier 3 decreased (from 66.8% to 60.7% for stroke and from 70.8% to 64.5% for IHD) over time (Table 2). In rural areas, however, the proportion of admissions in tier 1 and 2 hospitals decreased for stroke (from 39.4% to 26.5% in tier 1 and from 39.2% to 37.5% in tier 2) and IHD (from 48.1% to 38.0% in tier 1 and from 30.8% to 27.3% in tier 2), while the proportion of admissions in tier 3 increased (from 21.4% to 36.0% for stroke and from 21.1% to 34.6% for IHD) over the period. In CKB urban areas in 2009–2017, rates of hospitalisation increased by 12.30% per year in tier 1, 3.55% in tier 2 and decreased by 1.17% per year in tier 3 hospitals for stroke and increased by 5.65%, 3.63% and 2.24% per year for IHD, respectively (Appendix eTable 2). In CKB rural areas in 2009–2017, rates of hospitalisation for stroke did not change in tier 1 and tier 2 hospitals and increased by 12.5% in tier 3 hospitals, and for IHD, the rates of hospitalisation increased by 2.11% per year in tier 1, remained unchanged in tier 2 and increased by 13.20% per year in tier 3 hospitals.

**Table 2:** Proportions of admissions for stroke and ischaemic heart disease (IHD), reimbursement rates (RR) and deductibles (DD) by hospital tier in 2009 and 2017.

	2009				2017				% change between 2009 and 2017			
	Proportions of admissions (%)		Average RR (%)	Average DD (¥)	Proportions of admissions (%)		Average RR (%)	Average DD (¥)	Proportions of admissions (%)		Average RR	Average DD
	Stroke (n = 3251)	IHD (n = 2542)			Stroke (n = 5530)	IHD (n = 4278)			Stroke	IHD		
Urban areas												
Tier 1	5.0	6.5	90	509	7.5	9.1	91	325	50.0	39.2	1.2	−36.1
Tier 2	28.2	22.7	88	685	31.8	26.4	88	545	12.9	16.4	0.0	−20.4
Tier 3	66.8	70.8	86	872	60.7	64.5	86	738	−9.2	−8.9	−0.1	−15.4
Rural areas												
Tier 1	39.4	48.1	77	253	26.5	38.0	87	198	−32.8	−20.9	13.2	−21.7
Tier 2	39.2	30.8	66	539	37.5	27.3	77	416	−4.2	−11.3	15.6	−22.8
Tier 3	21.4	21.1	59	835	36.0	34.6	67	797	68.1	64.1	13.0	−4.6

Health insurance characteristics for stroke presented in table; health insurance characteristics for ischaemic heart disease (not presented) were similar. Reimbursement rates and deductibles were averaged across China Kadoorie Biobank (CKB) participants with first stroke by health insurance scheme and region, within urban and rural areas. These averages were then weighted by the proportion of CKB participants with first stroke enrolled in each scheme in urban and rural areas. Deductibles in 2009 were first standardised to 2017 values using consumer prices indices from the China statistical yearbooks.

### Impact of health insurance characteristics on choice of hospital tier

#### Urban areas

**Fig. 2:**
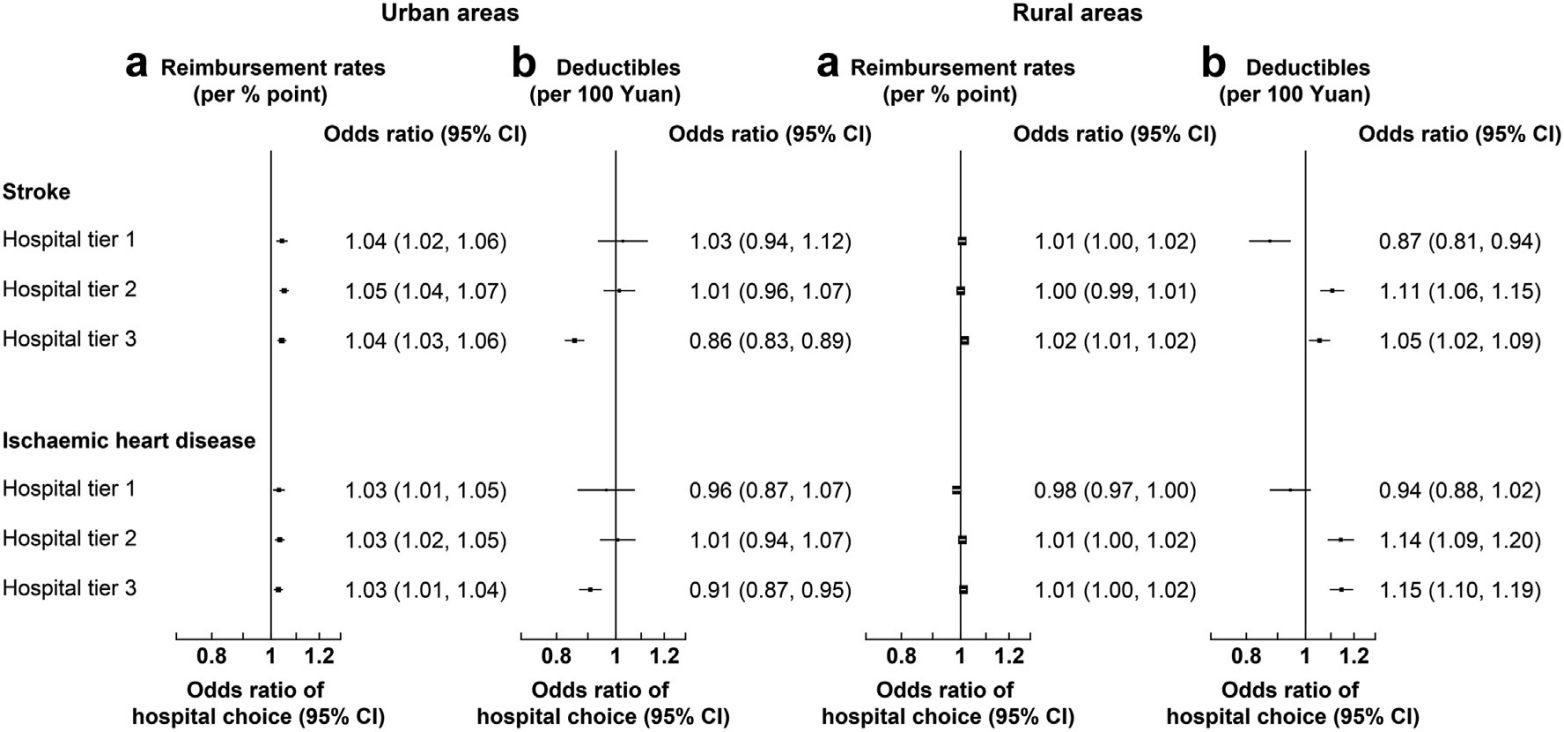
**Associations between higher reimbursement rates (a) or deductibles (b) and choice of hospital tier for stroke and ischaemic heart disease admissions in urban and rural areas**. Results are from multinomial logit models with the following tier-specific variables: interactions between tier and reimbursement rate and deductible, respectively. Only tier-specific odds ratios are presented and interpreted as follows: an odds ratio > 1 means that if the regressor increases for one hospital tier, then that tier is chosen more; and vice versa for an odds ratio < 1. Case-specific relative rate ratios from these models are presented in Appendix eTables 4 and 6.

Higher reimbursement rates in all tiers were associated with a higher likelihood of choosing the corresponding hospital tiers and higher deductibles in tier 3 hospitals were associated with a lower likelihood of choosing tier 3 hospitals (Fig. 2).

**Fig. 3:**
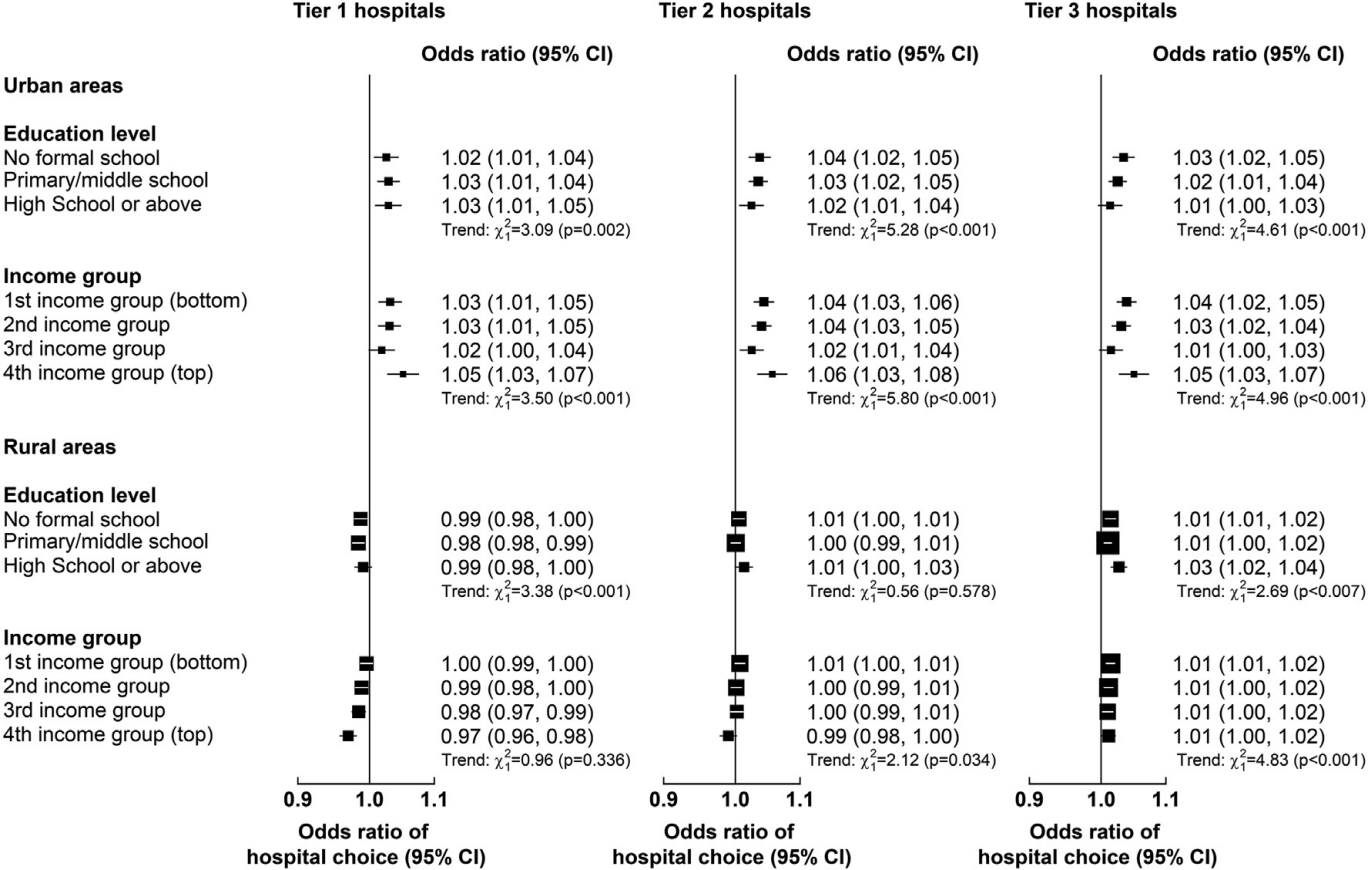
**Associations (Odds Ratios [95% CI]) between reimbursement rates (per % point) and choice of hospital tier for admissions for stroke and IHD combined in urban and rural areas, by income and education level.** Results are from multinomial logit models with the following tier-specific variables: reimbursement rate and deductible for each hospital tier by income or education level. Case-specific variables included demographic, socioeconomic (excluding income or education), lifestyle and morbidity factors, and log reimbursement ceiling. Only tier-specific odds ratios are presented and interpreted as follows: an odds ratio > 1 means that if the regressor increases for one hospital tier, then that hospital tier is chosen more; and vice versa for an odds ratio < 1.

For admissions with stroke, a 1% point higher reimbursement rate was associated with a 4% (OR: 1.04, 95% CI [1.02, 1.06]), 5% (1.05 [1.04, 1.07]) and 4% (1.04 [1.03, 1.06]) higher odds of choosing tier 1, 2 and 3 hospitals, respectively. For admissions with IHD, a 1% point higher reimbursement rate was associated with 3% higher odds of choosing respective hospital tier. The impact of tier 2 or 3 reimbursement rates was greater for individuals with lower education or income levels (Fig. 3). In post-estimation analysis, lowering tier 3 reimbursement rates by 5% was associated with a 3.25% points lower probability of using tier 3 hospitals for stroke and a 1.86% points lower probability for IHD, and a 0.51% and 2.75% points higher probability of using tier 1 and tier 2 hospitals for stroke and 0.42% and 1.86% points for IHD, respectively (Appendix eTable 3). Overall, the choice of hospital tier was elastic (range: 1.09–3.13) to changes in reimbursement rates (Table 3). For example, a 1% higher proportional increase in the reimbursement rate in tier 1 and 2 hospitals was associated with a greater increase in same tier choice probabilities for stroke (3.13% and 2.55%, respectively) and IHD (2.34% and 1.91%).

**Table 3:** Own and cross elasticities (95% CI) of choice of hospital tier for stroke and IHD in urban areas.

With respect to:	Stroke			IHD		
	Tier 1	Tier 2	Tier 3	Tier 1	Tier 2	Tier 3
Reimbursement rates						
Tier 1	3.13 (1.61, 4.62)	−0.28 (−0.42, −0.14)	−0.17 (−0.26, −0.09)	2.34 (0.73, 3.95)	−0.18 (−0.31, −0.06)	−0.1 (−0.24, −0.04)
Tier 2	−1.64 (−2.17, −1.10)	2.55 (1.73, 3.37)	−1.09 (−1.45, −0.74)	−0.85 (−1.27, −0.42)	1.91 (0.96, 2.84)	−0.66 (−0.99, −0.33)
Tier 3	−1.68 (−2.29, −1.06)	−1.79 (−2.43, −1.13)	1.00 (0.64, 1.36)	−1.43 (−2.20, −0.63)	−1.43 (−2.20, −0.63)	0.66 (0.29, 1.01)
Deductibles						
Tier 1	0.06 (−0.16, 0.28)	−0.01 (−0.03, 0.01)	0.00 (−0.01, 0.01)	−0.08 (−0.33, 0.17)	0.01 (−0.01, 0.03)	0.01 (−0.01, 0.02)
Tier 2	−0.03 (−0.13, 0.09)	0.04 (−0.13, 0.20)	−0.02 (−0.08, 0.06)	−0.01 (−0.10, 0.08)	0.02 (−0.18, 0.23)	−0.01 (−0.08, 0.06)
Tier 3	0.50 (0.38, 0.62)	0.54 (0.42, 0.66)	−0.31 (−0.38, −0.24)	0.38 (0.21, 0.54)	0.38 (0.21, 0.55)	−0.18 (−0.26, −0.10)

Elasticities were based on the effects of the reimbursement rates and deductibles on choice of hospital tier estimated in the main analysis (c.f. Fig. 1).

Example for stroke: a 1% increase in the reimbursement rate in tier 3 is associated with: a 1.00% increase in the probability of choosing tier 3 hospitals (own elasticity), a 1.68% and 1.79% decrease in the probability of choosing tier 1 and 2 hospitals, respectively (cross elasticities). Choice elasticity is elastic when |elasticity| > 1 and inelastic when |elasticity| < 1.

**Fig. 4:**
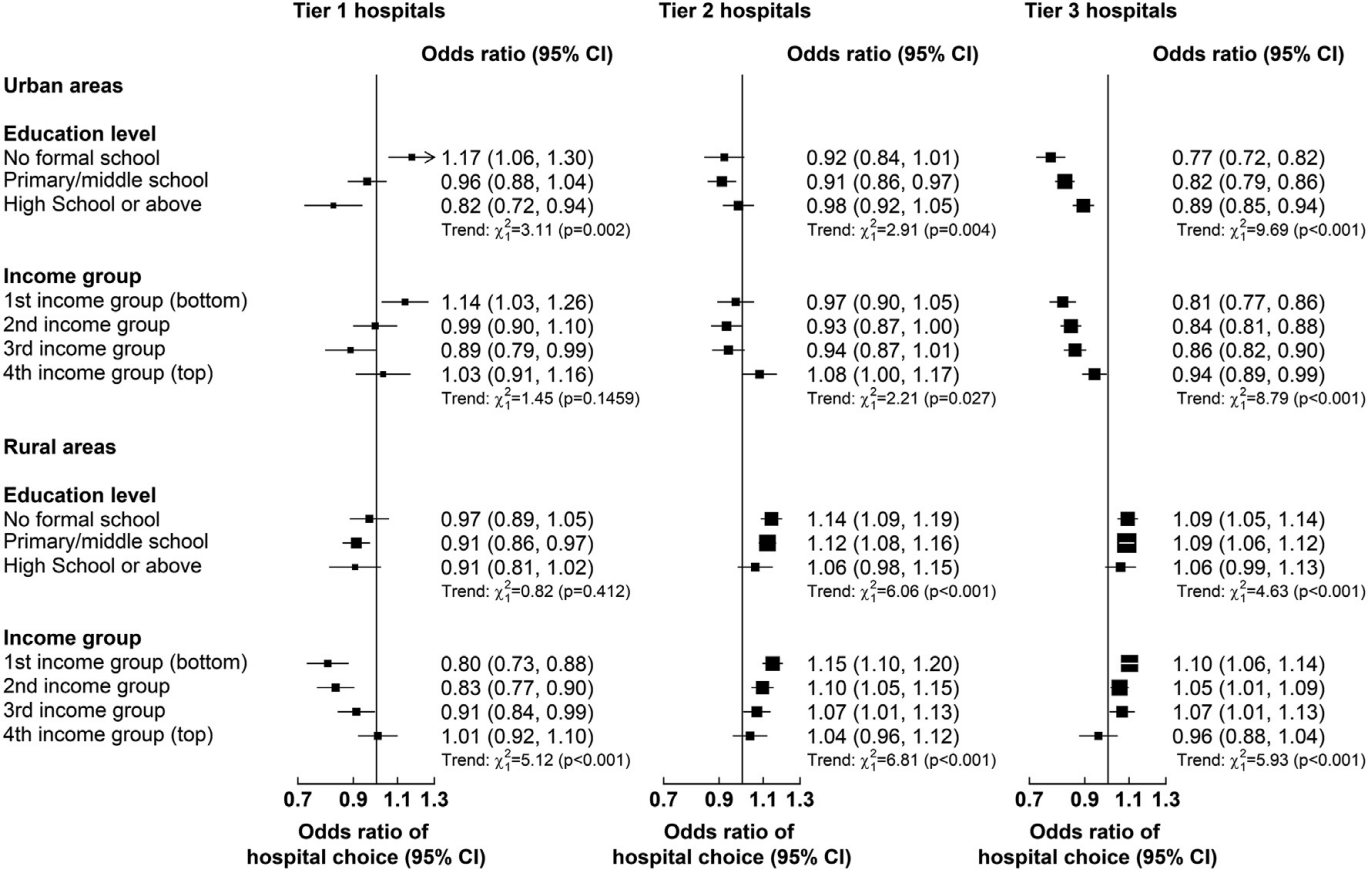
**Associations (Odds Ratios [95% CI]) between deductibles (per 100 Yuan) and choice of hospital tier for admissions for stroke and IHD combined in urban and rural areas, by income and education level.** Results are from multinomial logit models with the following tier-specific variables: reimbursement rate and deductible for each hospital tier by income or education level. Case-specific variables included demographic, socioeconomic (excluding income or education), lifestyle and morbidity factors, and log reimbursement ceiling. Only tier-specific odds ratios are presented and interpreted as follows: an odds ratio > 1 means that if the regressor increases for one hospital tier, then that hospital tier is chosen more; and vice versa for an odds ratio < 1.

A 100¥ higher deductible in tier 3 was associated with 14% (0.86 [0.83, 0.89]) and 9% (0.91 [0.87, 0.95]) lower odds of choosing tier 3 hospitals for stroke and IHD, respectively (Fig. 2). The impact of higher tier 3 deductibles was stronger for individuals with lower income or education levels in urban areas (Fig. 4). Overall, the choice of hospital tier was inelastic (range: 0.18–0.54) to changes in tier 3 deductibles (Table 3). A 10% higher ceiling was associated with 0.47% lower relative risk of choosing tier 2 (vs tier 3) hospitals for stroke (Appendix eTable 4).

#### Rural areas

Higher reimbursement rates in tier 3 hospitals were associated with a somewhat higher likelihood of choosing these hospitals and higher deductibles in tier 1 hospitals were associated with a lower likelihood of choosing these hospitals (Fig. 2). Associations between deductibles in tier 2 or 3 and choice of hospital tier were not in the expected direction.

A 1% point higher reimbursement rate in tier 3 was associated with a 2% (1.02 [1.01, 1.02]), and 1% (1.01 [1.00, 1.02]) higher odds of choosing tier 3 for individuals with stroke and IHD, respectively (Fig. 2). In post-estimation analysis, a 5% lower tier 3 reimbursement rate was associated with an 0.84% points lower probability of using tier 3 hospitals for stroke and a 0.58% points lower probability for IHD (Appendix eTable 5). Generally, choice of hospital tier for stroke and IHD in rural areas was inelastic to changes in reimbursement rates (range: 0.14–0.58; Table 4).

**Table 4:** Own and cross elasticities (95% CI) of choice of hospital tier for stroke and IHD in rural areas.

With respect to:	Stroke			IHD		
	Tier 1	Tier 2	Tier 3	Tier 1	Tier 2	Tier 3
Reimbursement rates						
Tier 1	0.27 (−0.22, 0.76)	−0.12 (−0.33, 0.09)	−0.11 (−0.32, 0.09)	−0.58 (−1.07, −0.09)	0.53 (0.08, 0.95)	0.51 (0.08, 0.93)
Tier 2	−0.01 (−0.21, 0.19)	0.01 (−0.30, 0.34)	−0.01 (−0.25, 0.23)	−0.11 (−0.25, 0.03)	0.33 (−0.10, 0.78)	−0.14 (−0.32, 0.04)
Tier 3	−0.22 (−0.30, −0.13)	−0.26 (−0.36, −0.15)	0.57 (0.34, 0.80)	−0.14 (−0.23, −0.06)	−0.19 (−0.31, −0.07)	0.44 (0.17, 0.70)
Deductibles						
Tier 1	−0.13 (−0.21, −0.05)	0.06 (0.02, 0.09)	0.05 (0.02, 0.09)	−0.04 (−0.11, 0.02)	0.04 (−0.02, 0.10)	0.04 (−0.02, 0.10)
Tier 2	−0.15 (−0.21, −0.08)	0.23 (0.13, 0.33)	−0.17 (−0.24, −0.10)	−0.12 (−0.16, −0.08)	0.40 (0.27, 0.53)	−0.17 (−0.23, −0.11)
Tier 3	−0.10 (−0.17, −0.03)	−0.12 (−0.20, −0.03)	0.26 (0.07, 0.44)	−0.23 (−0.30, −0.16)	−0.31 (−0.41, −0.22)	0.71 (0.49, 0.92)

Elasticities were based on the effects of the reimbursement rates and deductibles on choice of hospital tier estimated in the main analysis (c.f. Fig. 1). Example for stroke: a 1% increase in the reimbursement rate in tier 3 is associated with: a 0.57% increase in the probability of choosing tier 3 hospitals (own elasticity), a 0.22% and 0.26% decrease in the probability of choosing tier 1 and 2 hospitals, respectively (cross elasticities). Choice elasticity is elastic when |elasticity| > 1 and inelastic when |elasticity| < 1. Confidence intervals were estimated using 10,000 runs of the prediction on sets of random draws of model coefficients, and then taking the 2.5th and 97.5th percentiles for the 95% confidence interval. Elasticities can be computed from the changes in probabilities of hospital tier choice in Appendix eTable 3 (% change in choice probability/% change in reimbursement rate or deductible). For example, using results from Appendix eTable 3, the own elasticity for reimbursement rate in Tier 3 for stroke is approximately (0.85/29.42)/0.05 = 0.58 (∼0.57).

A 100¥ higher deductibles in tier 1 was associated with 13% (0.87 [0.81, 0.94]) and 6% (0.94 [0.88, 1.02]) lower odds of choosing tier 1 hospitals for stroke and IHD, respectively (Fig. 2). The impact of tier 1 deductibles was greater for individuals in lower income groups (Fig. 4). In addition, higher levels of deductibles in tier 2 or 3 hospitals were associated with a higher likelihood of choosing these hospitals for stroke and IHD, and the strength of these associations was greater for individuals with lower levels of education or income (Fig. 4). Generally, the choice of hospital tier for stroke and IHD in rural areas was inelastic to changes in deductibles (range: 0.05–0.71; Table 4). A 10% higher ceiling was associated with a 0.40% and 0.50% higher relative risks of choosing tier 1 and tier 2 (vs tier 3) hospitals for stroke, respectively (Appendix eTable 4).

### Relevance of individual characteristics for choice of hospital tier

**Fig. 5:**
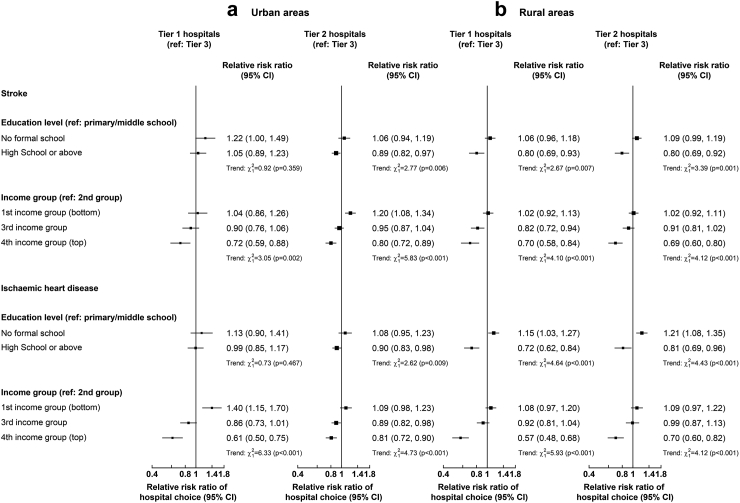
**Relevance of socioeconomic characteristics to choice of hospital tier for stroke and ischaemic heart disease admissions in urban (a) and rural (b) areas.** Results are from multinomial logit models with the following tier-specific variables: reimbursement rate and deductible for each hospital tier. Only selected case-specific relative risk ratios are presented and interpreted as parameters of a binary logit model against the base category “Tier 3 hospital”. Tier-specific odds ratios from these models are presented in Fig. 2 and other included case-specific variables, including demographic factors, other socioeconomic factors, lifestyle and morbidity factors and reimbursement ceiling, are presented in Appendix eTables 4 and 6.

In both urban and rural areas, compared with a choice of tier 3 hospitals as a reference, older individuals with stroke were more likely to choose tier 1 or 2 hospitals than younger individuals (Appendix eTable 4). Women were more likely to choose tier 1 or 2 hospitals for IHD (Appendix eTable 6). A higher household size was also associated with a higher likelihood of choosing tier 1 hospitals for IHD. Individuals with higher levels of education were less likely to choose tier 1 or 2 for stroke and IHD than those with lower educational levels (Fig. 5). Individuals with higher income were less likely to choose tier 1 or 2 hospitals for stroke and IHD than those with lower income. Compared to individuals with non-lacunar IS, those with lacunar IS were more likely to choose tier 1 or tier 2 than tier 3 hospitals, while those with HS were less likely to choose tier 1 or tier 2 (Appendix eTable 4). Individuals with other IHD were more likely to choose tier 1 or tier 2 than tier 3 hospitals than those with AMI or angina (Appendix eTable 6).

Furthermore, in urban areas only, women were less likely than men to choose tier 2 hospitals for stroke and higher household size was associated with a higher likelihood of choosing tier 2 hospitals for stroke. In rural areas only, older individuals with IHD were more likely to choose tier 2 hospitals, and individuals who were not married were more likely than married individuals to choose tier 1 for stroke and IHD.

### Sensitivity analyses

The associations between reimbursement rates and deductibles, and choice of hospital tier in minimally-adjusted models were similar to their associations in fully-adjusted models. When including reimbursement rates and deductibles separately rather than together, the effects of reimbursement rates were attenuated and the effects of deductibles were stronger and significant across all tiers for stroke, and similar to the ones in the main analysis for IHD. Excluding the reimbursement ceiling did not affect results. Models with combined effect for reimbursement rates and deductibles across hospital tiers had worse model fit than those with tier-specific reimbursement rates and deductibles.

In urban areas, higher OOP payments were associated with lower odds of choosing tier 2 and 3 hospitals and higher ARR were associated with higher odds of choosing tier 2 and 3 for stroke only (Appendix eTable 7). In rural areas, higher ARR and lower OOP payments were associated with higher odds of choosing tier 3 for stroke and tier 2 and 3 hospitals for IHD. The associations between reimbursement rates and deductibles, and choice of hospital tier were not affected by adjustments for regional supply-side factors. The number of beds was not significantly associated with choice of hospital tier and a higher density of doctors was associated with lower odds of choosing tier 1 or 2 (vs tier 3) hospitals in urban areas. For hospital admissions for any cause, the associations between HI and socioeconomic characteristics and choice of hospital tier in both urban and rural areas were similar to those for stroke and IHD, with somewhat stronger associations for tier 1 and 2 deductibles in urban areas (ORs per 100¥ higher deductibles: 0.91 [0.87, 0.95] and 0.93 [0.91, 0.96], respectively).

## Discussion

This study provides new evidence on the associations between health insurance cost-sharing, defined by levels of reimbursement rates and deductibles, and choice of hospital tiers for patients with first hospital admission for stroke and IHD in urban and rural areas in China. About 60% of study participants in urban areas and only 30% of participants in rural areas chose tier 3 hospitals for first stroke or IHD admissions in 2009–2017. The study reports that the estimated associations between health insurance cost-sharing parameters and choice of hospital tiers differed between urban and rural areas. Higher reimbursement rates and lower deductibles in lower tier hospitals may have contributed to the more limited increase in admissions in tier 3 hospitals compared to lower tier hospitals in the urban areas but is unlikely to have contributed substantively in rural areas where large increases in admissions to tier 3 hospitals were observed. In both urban and rural areas, individuals with higher socioeconomic status or greater disease severity were more likely to seek treatment in higher tier hospitals.

The study was motivated by established hypotheses originating in the economic theory of cost-sharing, namely that higher HI cost-sharing (lower reimbursement rates and higher deductibles) in a given hospital tier decreases the likelihood of choosing this hospital tier. However, the findings of the present study differed between urban and rural areas. The positive associations between reimbursement rates and choices of respective hospital tier in urban areas and choice of tier 3 hospitals in rural areas were consistent with the study hypothesis. For the deductibles, only associations for deductibles in tier 3 hospitals in urban areas and in tier 1 hospitals in rural areas were consistent with the study hypothesis. The remaining associations for reimbursement rates and deductibles in rural areas did not support the study hypotheses. Urban residents, particularly urban employees, may have been more familiar with the concept of insurance or better informed about differences in reimbursement policies than rural residents, as HI schemes have been implemented earlier than those in rural areas.^25^, ^26^, ^27^, ^28^ The somewhat surprising finding in rural areas of higher tier 2 and 3 deductibles being associated with higher use of these hospital tiers for stroke and IHD should not be over interpreted in view of the reported inelastic relationships between deductibles and choice of hospital tier. Tier 1 hospitals, especially in rural areas, frequently lack appropriate equipment and facilities for diagnosis and treatment and their quality of care is perceived as low,^29^ a perception likely exacerbated for more severe conditions such as stroke and IHD. In this context, higher deductibles in higher tier hospitals may be perceived as an indicator of higher quality of care and,^30^ together with our inability to adjust for quality of care, may have produced counterintuitive associations.^30^

The findings of the present study could inform HI policies and healthcare reforms that promote the appropriate and efficient use of hospital services. Greater differences in reimbursement rates and deductibles between hospital tiers, especially in urban areas, could enhance the use of lower tier hospitals for stroke and IHD patients. For example, differentiated reimbursement rates by disease type, with higher reimbursement rates in lower tier hospitals and lower reimbursement rates in higher tier hospitals for less severe diseases could be considered. Importantly, to encourage patients with less severe cardiovascular disease types to use lower tier hospitals, their trust in quality of services provided in these hospitals needs to be improved. This may require recruitment of additional physicians and nurses in tier 1 hospitals and more efficient transfers or referrals to higher tier hospitals for more specialised care. Improved quality of care in lower tier facilities could also strengthen trust in primary healthcare to function as a gate-keeper and guide patients to appropriate care for their illness. Moreover, local governments could improve communication with patients about their HI benefits, especially in rural areas and among lower socioeconomic groups who have limited access to this information and may have higher levels of mistrust in the HI schemes.^25^

Preferences for tier 3 hospitals by individuals in higher socioeconomic groups (i.e. higher income or education levels) and for tier 1 hospitals by individuals in the lowest socioeconomic groups are consistent with findings from previous studies and suggested that lower socioeconomic groups were more price-sensitive.^31^ A previous report indicated that distrust in primary care facilities was greater among patients with higher education.^32^ The preferences of older patients in rural areas for tier 1 or 2 (vs tier 3) hospitals compared to younger patients might reflect their lower socioeconomic status, preference for shorter travel distance or waiting times or provision of rehabilitation services in lower tier hospitals.^11^^,^^16^ The preference for care in tier 3 hospitals for patients with more severe diseases, and more adverse lifestyle factors, was consistent with findings from previous studies,^11^^,^^33^ reflecting their greater capacity for treatment of acute diseases or increased risk aversion among people with more severe diseases. Overall, the differential impacts of socioeconomic characteristics on the choice of hospital tiers highlights inequalities in access to different hospital tiers in China.

The chief strengths of the present study include the use of detailed annual data on HI characteristics for all social HI schemes, the use of HI claims data for choice of all three hospital tiers, the diversity in regions included, the extensive data collected on individual participants and the focus on inpatient hospital care for specific high-burden diseases and their subtypes. However, the study also had several limitations. Firstly, the use of HI claims data meant that data for hospitalised patients only were available and people not seeking treatment in hospitals were not included. However, the proportion of people not seeking care for major diseases such as stroke and IHD is likely small in the context of very high HI coverage during the study period. In addition, we were unable to assess the appropriateness of a given hospital tier for treatment of a particular stroke or IHD type. Secondly, our analyses were also constrained by unavailability of data in the CKB study on travel time and cost of travel to hospitals; patients' perception of quality of care in different hospital tiers; their attitudes to health risks; and possible other policy changes in 2009–2017, all of which may have introduced omitted variable bias.^30^ In this context, the hospital tiers classification and the reported associations may also reflect some of these missing factors. Information on participants’ critical illness insurance, medical financial assistance or private HI was also not available in CKB but their impact on study findings is likely more limited. The present study also did not adjust for policies related to the hierarchical medical system. While we did not have precise information for the implementation of the hierarchical medical system in CKB study areas, based on a study by Zhou et al. (2021), its implementation started in four of the provinces where CKB participants were recruited from in 2015, in two provinces in 2016 and in three provinces in 2017.^15^ Thus, it is unlikely that in its initial stages of implementation this policy would have had substantive effects across CKB areas. Thirdly, the socioeconomic and lifestyle factors were only collected at entry into the CKB study. However, as the study population is middle-aged and older, education is unlikely to change further, and although income has likely increased during the study period, relative income or categories of income are expected to remain stable over time. Future studies should include measurements of patient travel time and travel costs to hospitals, indicators for quality of care, and supply-side factors over time to enhance assessment of the associations between HI cost-sharing and choice of hospital tiers. Finally, the present study is an observational study and further randomised trials are required to clarify the causal effects of healthcare reforms.

In conclusion, changes in the reimbursement rates and deductibles for use of hospitals of different tiers could divert patients from higher to lower tier hospitals and restrain growth in healthcare costs. However, such changes may adversely affect health outcomes by reducing the use of appropriate healthcare, particularly among socially disadvantaged groups in the population, highlighting the need for a referral system based on medical need. To reduce rural-urban differences in hospital care, improvements are required to enhance access to skilled health professionals, diagnostic investigations and specialised healthcare in rural areas. Improvements in HI coverage for lower socioeconomic groups could also help reduce the socioeconomic disparities in the choice of hospital tiers. The study results add to the body of evidence on differential HI cost-sharing suggesting varying potential depending on local circumstances with respect to evolving health needs, healthcare provision and individual circumstances.

## Contributors

Study concept and design: Muriel Levy, John Buckell, Robert Clarke, Yiping Chen, Winnie Yip and Borislava Mihaylova. Data collection and quality control: Muriel Levy, Nina Wu, Pei Pei, Dianjianyi Sun, Daniel Avery, Hua Zhang, Jun Lv, Canqing Yu, Liming Li, Zhengming Chen, Winnie Yi and Yiping Chen. Data analysis and interpretation: Muriel Levy, John Buckell, Daniel Avery and Borislava Mihaylova. Manuscript draft: Muriel Levy. Manuscript review and editing: John Buckell, Robert Clarke, Winnie Yip, Yiping Chen and Borislava Mihaylova. All authors read and approved the final manuscript.

## Data sharing statement

The China Kadoorie Biobank (CKB) is a global resource for the investigation of lifestyle, environmental, blood biochemical and genetic factors as determinants of common diseases. The CKB study group is committed to making the cohort data available to the scientific community in China, the UK and worldwide to advance knowledge about the causes, prevention and treatment of disease. For detailed information on what data is currently available to open access users and how to apply for it, visit: https://www.ckbiobank.org/data-access.

Researchers who are interested in obtaining the raw data from the China Kadoorie Biobank study that underlines this paper should contact ckbaccess@ndph.ox.ac.uk. A research proposal will be requested to ensure that any analysis is performed by bona fide researchers and–where data is not currently available to open access researchers–is restricted to the topic covered in this paper.

## Declaration of interests

All authors declare no competing interests.
