# Supplementary material for: Association between health insurance cost-sharing and choice of hospital tier for cardiovascular diseases in China: a prospective cohort study
Source: Lancet Reg Health West Pac. 2024 Feb 14;45:101020. doi: 10.1016/j.lanwpc.2024.101020 (PMC10876671; doi:10.1016/j.lanwpc.2024.101020)
Supplement: Appendix Material [file mmc1.docx]

**Appendix Material**

**Association between health insurance cost-sharing and choice of hospital tier for cardiovascular diseases in China: a prospective cohort study**

**Table of Contents**

| Page |  |
| --- | --- |
| 2 | Table of contents |
| 3 | Members of the China Kadoorie Biobank Collaborative Group |
| 4 | Appendix Method A: Description of the healthcare system in China |
| 4 | Appendix Method B: Description of conditional logit choice models |
| 5 | Appendix Method C: Description of the selection of the conditional logit choice model |
| 5 | Appendix Method D: Description of the method used to calculate out-of-pocket (OOP) payments and actual reimbursement rates (ARR) |
| 6 | eTable 1: Reimbursement rates and deductibles for the main health insurance scheme in each CKB area in 2009 and 2017 |
| 7 | eTable 2: Fully adjusted* annual percentage change (95% CI) in rates of hospitalisation for stroke and IHD by hospital tier in urban and rural areas in 2009-2017 |
| 8 | eTable 3: Effects (95% CI) of changes in reimbursement rates and deductibles on probabilities of hospital tier choice for stroke and IHD in urban areas |
| 9 | eTable 4: Relevance of patient characteristics and reimbursement ceiling (Relative rate ratios [95% CI]) to hospital tier choice for stroke admissions |
| 11 | eTable 5: Effects (95% CI) of changes in reimbursement rates and deductibles on probabilities of hospital tier choice for stroke and IHD in rural areas |
| 12 | eTable 6: Relevance of patient characteristics and reimbursement ceiling (Relative rate ratios [95% CI]) to hospital tier choice for IHD admissions |
| 14 | eTable 7: Associations (Odds Ratios [95% CI]) between out-of-pocket (OOP) payments or actual reimbursement rates (ARR) and choice of hospital tier for admissions for stroke and IHD |

**Members of the China Kadoorie Biobank Collaborative Group**

***International Steering Committee:*** Junshi Chen, Zhengming Chen (PI), Robert Clarke, Rory Collins, Liming Li (PI), Chen Wang, Jun Lv, Richard Peto, and Robin Walters.

***International Co-ordinating Centre, Oxford:*** Daniel Avery, Maxim Barnard, Derrick Bennett, Ruth Boxall, Kahung Chan, Yiping Chen, Zhengming Chen, Johnathan Clarke, Robert Clarke, Huaidong Du, Ahmed Edris Mohamed, Hannah Fry, Simon Gilbert, Pek Kei Im, Andri Iona, Maria Kakkoura, Christiana Kartsonaki, Hubert Lam, Kuang Lin, James Liu, Mohsen Mazidi, Iona Millwood, Sam Morris, Qunhua Nie, Alfred Pozaricki, Paul Ryder, Saredo Said, Dan Schmidt, Becky Stevens, Iain Turnbull, Robin Walters, Baihan Wang, Lin Wang, Neil Wright, Ling Yang, Xiaoming Yang and Pang Yao.

***National Co-ordinating Centre, Beijing:*** Xiao Han, Can Hou, Qingmei Xia, Chao Liu, Jun Lv, Pei Pei, Dianjianyi Sun, Canqing Yu.

***Regional Co-ordinating Centres:*** **Guangxi** Provincial CDC: Naying Chen, Duo Liu, Zhenzhu Tang. Liuzhou CDC: Ningyu Chen, Qilian Jiang, Jian Lan, Mingqiang Li, Yun Liu, Fanwen Meng, Jinhuai Meng, Rong Pan, Yulu Qin, Ping Wang, Sisi Wang, Liuping Wei, Liyuan Zhou. **Gansu** Provincial CDC: Caixia Dong, Pengfei Ge, Xiaolan Ren. Maiji CDC: Zhongxiao Li, Enke Mao, Tao Wang, Hui Zhang, Xi Zhang. **Hainan** Provincial CDC: Jinyan Chen, Ximin Hu, Xiaohuan Wang. Meilan CDC: Zhendong Guo, Huimei Li, Yilei Li, Min Weng, Shukuan Wu. **Heilongjiang** Provincial CDC: Shichun Yan, Mingyuan Zou, Xue Zhou. Nangang CDC: Ziyan Guo, Quan Kang, Yanjie Li, Bo Yu, Qinai Xu. **Henan** Provincial CDC: Liang Chang, Lei Fan, Shixian Feng, Ding Zhang, Gang Zhou. Huixian CDC: Yulian Gao, Tianyou He, Pan He, Chen Hu, Huarong Sun, Xukui Zhang. **Hunan** Provincial CDC: Biyun Chen, Zhongxi Fu, Yuelong Huang, Huilin Liu, Qiaohua Xu, Li Yin. Liuyang CDC: Huajun Long, Xin Xu, Hao Zhang, Libo Zhang. **Jiangsu** Provincial CDC: Jian Su, Ran Tao, Ming Wu, Jie Yang, Jinyi Zhou, Yonglin Zhou. Suzhou CDC: Yihe Hu, Yujie Hua, Jianrong Jin, Fang Liu, Jingchao Liu, Yan Lu, Liangcai Ma, Aiyu Tang, Jun Zhang. **Qingdao** CDC: Liang Cheng, Ranran Du, Ruqin Gao, Feifei Li, Shanpeng Li, Yongmei Liu, Feng Ning, Zengchang Pang, Xiaohui Sun, Xiaocao Tian, Shaojie Wang, Yaoming Zhai, Hua Zhang, Licang CDC: Wei Hou, Silu Lv, Junzheng Wang. **Sichuan** Provincial CDC: Xiaofang Chen, Xianping Wu, Ningmei Zhang, Xiaoyu Chang. Pengzhou CDC: Xiaofang Chen, Jianguo Li, Jiaqiu Liu, Guojin Luo, Qiang Sun, Xunfu Zhong. **Zhejiang** Provincial CDC: Weiwei Gong, Ruying Hu, Hao Wang,Meng Wang, Min Yu. Tongxiang CDC: Lingli Chen, Qijun Gu, Dongxia Pan，Chunmei Wang, Kaixu Xie, Xiaoyi Zhang.

**Stroke Adjudication Committee**: Hongyuan Chen, Liyang Liu, Haiyan Gou, Xun Wang, Jing Ding, Ning Zhang, Yueshi Mao, Shanshan Zhou, Lirong Jin, Xin Cheng, Yun Lu, Li Chen, Zilong Hao, Xiaona Xing, Lei Wang, Naixin Ju, Yiting Mao, Shuya Li, Peng Du, Deren Wang, Xiaojia Sun, Shihao You, Weizhi Wang, Yanmei Zhu, Xiaojiu Li and Yi Dong.

**Appendix Method A: Description of the healthcare system in China**

*Hospital tiers*

Since the 1990s, hospitals in China are classified into tiers, based on their capacity, including number of beds, clinical departments, number of medical personnel, types of equipment, and the floor area per bed. Tier 3 hospitals include ministerial, provincial, municipal and teaching hospitals, that provide high-level and specialised medical services and are responsible for higher education and scientific research. Tier 3 hospitals have at least 500 beds and typically have close to 100% occupancy rate. Tier 2 hospitals are mainly county and district level hospitals, including traditional Chinese medicine hospitals, and provide comprehensive medical and health services to multiple communities and provide facilities for medical training and research. Tier 2 hospitals have between 100 and 500 beds and typically have about 90% occupancy rate. Tier 1 hospitals provide preventive services, clinical treatment, rehabilitation services and health education in the community, have between 20 and 99 beds and typically have about 64% occupancy rate. Tier 1 healthcare facilities include community health centres and stations in urban areas and township health centres and village clinics in rural areas, and are part of the primary care system.

*Health insurance (HI) schemes*

Three main social HI schemes operate in China: the Urban Employee Basic Medical Insurance (UEBMI), the New Rural Cooperative Medical Scheme (NRCMS) and the Urban Resident Basic Medical Insurance (URBMI). The HI schemes differ from each other in eligibility criteria, administration, source of funding and benefits. Enrolment in a specific HI scheme depends on an individual’s employment status and household registration status (hukou). Each scheme includes cost-sharing mechanisms for inpatient and outpatient care, including deductibles, reimbursement rates, and reimbursement ceiling. The deductible is the amount of healthcare costs paid by the insured person per admission or visit to receive medical care after which a reimbursement schedule is applied. The reimbursement rate is the percentage of total medical expenses minus the deductible paid by the HI scheme. The ceiling is the maximum amount paid by the insurance scheme in any year and is often at least four times the average income in the municipality or county. While being important contributors to overall cost, the deductible and coinsurance rate also aim to reduce unnecessary hospital care use. Local governments are given autonomy to specify the benefit packages, provider payment arrangements, and services covered by the HI schemes, based on the local economy and population needs.

**Appendix Methods B: Description of conditional logit choice models**

Conditional logit (McFadden’s) choice models were used to examine associations between HI cost-sharing and patient characteristics and choice of hospital tier. The choice of hospital tier was assumed to maximise an individual’s utility ($U$). That is, an individual $n$, will only choose a hospital tier $i$ if $U_{n,i}>U_{n,j} \forall j \neq i$. Utility was further decomposed to include a deterministic component $V_{n,i}$ and a random component $\varepsilon_{n,i}$ (error term): $U_{n,i}= V_{n,i}+ \varepsilon_{n,i}$. The error term $\varepsilon_{ni}$ measured deviation from modelled utility for hospital tier $i$ and individual $n$, and captured all factors not included in $V_{n,i}$. Thus, the probability that an individual $n$ chose tier $i$ was given by:

$P_{n,i}=Prob \left( V_{n,i}+ \varepsilon_{n,i}>V_{n,j}+\varepsilon_{n,j} \right) \forall j\neq i$.

In very general terms, $V_{n,i}=\delta_{i}+ f(x_{n,i} , z_{n}, \beta)$. $x_{n,i}$ were characteristics of tier $i$ as faced by individual $n$, also called alternative or tier-specific variables. $z_{n}$ were characteristics of individual $n$, also called case-specific variables. $\beta$ was a vector of estimated parameters. $\delta_{i}$ were tier-specific constants capturing the mean of the error terms across hospital tiers. The average effect on utility of all factors not included in the model were captured by the tier-specific constants. Assuming that each $\varepsilon_{ni}$ for all tiers $i$ is distributed independently and identically in accordance with the extreme value distribution, the probability that a patient will choose hospital tier $i$ was given by:

$$P_{n,i}=\frac{e^{V_{n,i}}}{\sum_{j=1}^{J} e^{V_{n,j}}}$$

**Appendix Method C: Description of the selection of the conditional logit choice model**

The multinomial logit embodies the “independence of irrelevant alternatives” (IIA) assumption. IIA implies that the choice between any two alternatives does not depend upon a third one, namely the ratio of choice probabilities for alternatives i and j does not depend on characteristics of other alternatives. Different tests, including the Hausman test, the “cross-alternative” method and the model-testing method (i.e. estimating nested logit and mixed logit models) were performed to check if the IIA was reasonable.(1) The results of the different tests were inconclusive and there was no evidence that the IIA assumption was inappropriate.

Furthermore, previous work indicated that IIA assumption was not realistic in many situations and that IIA assumption in the multinomial logit was not as restrictive as perceived,(2) and that choice set partitioning tests of IIA were not appropriate for applied work.(3)

**Appendix Method D: Description of the method used to calculate out-of-pocket (OOP) payments and actual reimbursement rates (ARR)**

OOP payments and ARR were approximated using annual data on medical expenses of discharged patients with stroke or IHD by hospital grade at the national level (see Table below).

Average medical expenses for AMI were used as a proxy for IHD as data on hospitalisation costs for IHD were not available.

OOP payments (Yuan per admission) and ARR (%) were calculated using the following formulas:

$\mathrm{OOP}_{\mathrm{irft}}=\mathrm{Deductible}_{\mathrm{irft}}+{(1-Reimbursement rate}_{\mathrm{irft}})*{(Cost}_{\mathrm{ft}}-\mathrm{Deductible}_{\mathrm{irft}})$, and

$$\mathrm{ARR}_{\mathrm{irft}}=1- \frac{\mathrm{OOP}_{\mathrm{irft}}}{\mathrm{Cost}_{\mathrm{ft}}}$$

with $i$: individual, $r$: region, $f$: calendar year, $t$: hospital tier

The reimbursement ceiling was ignored when calculating OOP payments, because it was assumed that patients were unlikely to reach the reimbursement ceiling in cases of first stroke or IHD admission.

| **Per capita medical expenses (Yuan) of discharged patients in 2009 and 2017, by hospital tier** | | | | | | |
| --- | --- | --- | --- | --- | --- | --- |
|  | **2009** | | | **2017** | | |
|  | **Tier 1** | **Tier 2** | **Tier 3** | **Tier 1** | **Tier 2** | **Tier 3** |
| **Ischaemic stroke** | 5277.0 | 6954.8 | 9598.7 | 6053.2 | 7838.7 | 12008.5 |
| **Haemorrhagic stroke** | 8465.5 | 11404.9 | 20405.4 | 13764.0 | 12452.8 | 21949.0 |
| **AMI** | 5467.6 | 11390.1 | 25592.5 | 12833.7 | 17735.6 | 30270.9 |
| **Any disease** | 3205.4 | 11404.9 | 11979.8 | 4602.8 | 5799.1 | 13086.7 |
| AMI: Acute myocardial infarction. Medical expenses in 2009 were inflated to 2017 values. Source: China health statistical yearbooks.(4) | | | | | | |

| **eTable 1: Reimbursement rates and deductibles for the main health insurance scheme in each CKB area in 2009 and 2017** | | | | | | | | | | | | | | |
| --- | --- | --- | --- | --- | --- | --- | --- | --- | --- | --- | --- | --- | --- | --- |
|  |  | **2009** | | | | | |  | **2017** | | | | | |
|  |  | **Reimbursement rate (%)** | | | **Deductible (Yuan)** | | |  | **Reimbursement rate (%)** | | | **Deductible (Yuan)** | | |
|  |  | **Tier 1** | **Tier 2** | **Tier 3** | **Tier 1** | **Tier 2** | **Tier 3** |  | **Tier 1** | **Tier 2** | **Tier 3** | **Tier 1** | **Tier 2** | **Tier 3** |
| **Urban areas** |  |  |  |  |  |  |  |  |  |  |  |  |  |  |
| **Qingdao** | UEBMI | 95 | 95 | 95 | 500 | 670 | 840 |  | 90 | 88 | 86 | 200 | 500 | 800 |
|  | URBMI | 70 | 70 | 70 | 500 | 670 | 840 |  | 85 | 80 | 70 | 200 | 500 | 800 |
| **Harbin** | UEBMI | 90 | 90 | 90 | 240 | 480 | 720 |  | 90 | 90 | 90 | 240 | 480 | 720 |
|  | Provincial | 94 | 91 | 85 | 240 | 480 | 720 |  | 94 | 91 | 85 | 240 | 480 | 720 |
|  | Railway | 79 | 72 | 65 | 240 | 480 | 720 |  | 81 | 75 | 65 | 240 | 480 | 720 |
|  | URBMI | 71 | 61 | 61 | 240 | 480 | 720 |  | 75 | 65 | 65 | 240 | 480 | 720 |
| **Haikou** | UEBMI | 88 | 88 | 85 | 800 | 800 | 800 |  | 88 | 85 | 82 | 300 | 600 | 800 |
|  | URBMI | 60 | 55 | 50 | 130 | 400 | 700 |  | 90 | 75 | 65 | 100 | 300 | 350 |
|  | NRCMS | 88 | 88 | 85 | 800 | 800 | 800 |  | 88 | 85 | 82 | 300 | 600 | 800 |
| **Suzhou** | UEBMI | 90 | 90 | 90 | 400 | 600 | 800 |  | 90 | 90 | 90 | 400 | 600 | 800 |
|  | URBMI/NRCMS | 70 | 70 | 70 | 400 | 600 | 800 |  | 75 | 75 | 75 | 200 | 400 | 600 |
| **Liuzhou** | UEBMI | 92 | 87 | 82 | 400 | 600 | 800 |  | 92 | 87 | 82 | 400 | 600 | 800 |
|  | URBMI | 60 | 50 | 40 | 150 | 300 | 500 |  | 90 | 75 | 60 | 200 | 400 | 600 |
|  | NRCMS | 80 | 50 | 40 | 50 | 150 | 200 |  | 90 | 75 | 60 | 200 | 400 | 600 |
| **Rural areas** | | | | | | | |  |  |  |  |  |  |  |
| **Sichuan** | NRCMS/URBMI | 80 | 65 | 50 | 100 | 200 | 500 |  | 85 | 75 | 50 | 100 | 200 | 500 |
|  | UEBMI | 92 | 87 | 82 | 400 | 600 | 800 |  | 92 | 87 | 82 | 400 | 600 | 800 |
| **Gansu** | NRCMS | 80 | 60 | 55 | 100 | 500 | 700 |  | 85 | 75 | 65 | 100 | 300 | 800 |
|  | UEBMI | 77 | 76 | 75 | 150 | 400 | 800 |  | 77 | 76 | 75 | 150 | 400 | 800 |
|  | URBMI | 85 | 75 | 55 | 150 | 350 | 700 |  | 85 | 75 | 65 | 100 | 300 | 700 |
| **Henan** | NRCMS | 65 | 55 | 40 | 80 | 300 | 800 |  | 90 | 75 | 72 | 200 | 500 | 900 |
|  | UEBMI | 87 | 86 | 85 | 300 | 450 | 800 |  | 88 | 85 | 82 | 300 | 450 | 1000 |
|  | URBMI | 50 | 60 | 70 | 200 | 400 | 600 |  | 87 | 80 | 75 | 300 | 400 | 1000 |
| **Zhejiang** | NRCMS/URBMI | 55 | 55 | 55 | 300 | 500 | 500 |  | 85 | 75 | 65 | 300 | 500 | 800 |
|  | UEBMI | 91 | 88 | 85 | 1000 | 1000 | 1000 |  | 90 | 85 | 80 | 300 | 500 | 800 |
| **Hunan** | NRCMS/URBMI | 80 | 65 | 55 | 100 | 300 | 500 |  | 85 | 70 | 50 | 200 | 500 | 800 |
|  | UEBMI | 96 | 95 | 92 | 480 | 650 | 900 |  | 95 | 91 | 88 | 480 | 650 | 900 |

| **eTable 2: Fully adjusted* rates of hospitalisation per 1000 person-years (95% CI) and annual percentage change (95% CI) for stroke and IHD by hospital tier in urban and rural areas in 2009 and 2017** | | | | | |
| --- | --- | --- | --- | --- | --- |
|  |  | **Stroke** | | **IHD** | |
|  |  | **Urban areas** | **Rural areas** | **Urban areas** | **Rural areas** |
| **Tier 1** | 2009 | 0.71 (0.60, 0.83) | 4.01 (3.75, 4.26) | 0.96 (0.82, 1.11) | 3.59 (3.32, 3.86) |
|  | 2017 | 2.21 (2.04, 2.38) | 4.05 (3.85, 4.25) | 1.42 (1.28, 1.55) | 3.43 (3.24, 3.62) |
|  | Annual % change | 12.3 (10.7, 13.8) | -0.02 (-0.75, 0.72) | 5.65 (3.80, 7.54) | 2.11 (1.31, 2.92) |
| **Tier 2** | 2009 | 2.87 (2.60, 3.13) | 3.05 (2.83, 3.27) | 2.35 (2.11, 2.59) | 1.47 (1.30, 1.64) |
|  | 2017 | 4.72 (4.47, 4.96) | 2.68 (2.52, 2.83) | 3.21 (2.98, 3.44) | 1.53 (1.41, 1.66) |
|  | Annual % change | 3.55 (2.44, 4.67) | -0.43 (-1.25, 0.39) | 3.63 (2.38, 4.89) | -0.32 (-1.69, 1.07) |
| **Tier 3** | 2009 | 11.04 (10.54, 11.54) | 1.33 (1.19, 1.48) | 9.84 (9.37, 10.32) | 1.15 (1.01, 1.29) |
|  | 2017 | 10.18 (9.81, 10.56) | 3.14 (2.97, 3.31) | 11.84 (11.4, 12.27) | 2.96 (2.77, 3.16) |
|  | Annual % change | -1.17 (-1.73, -0.60) | 12.5 (11.4, 13.5) | 2.24 (1.66, 2.83) | 13.2 (12.0, 14.5) |
| *The fully adjusted models included adjustments for demographic factors (age and sex), socioeconomic factors (marital status, household size, education, income, and health insurance type), lifestyle factors (smoking, alcohol consumption, body mass index, physical activity, self-reported health), morbidity factors, and region. | | | | | |

| **eTable 3: Effects (95% CI) of changes in reimbursement rates and deductibles on probabilities of hospital tier choice for stroke and IHD in urban areas** | | | | | | | |  |
| --- | --- | --- | --- | --- | --- | --- | --- | --- |
|  | | **Stoke** | | | **IHD** | | | |
|  | | **Tier 1** | **Tier 2** | **Tier 3** | **Tier 1** | **Tier 2** | **Tier 3** | |
| **Baseline probabilities (%)** | | 6.03  (0.34, 24.62) | 30.44  (5.69, 64.82) | 63.53  (22.75, 93.55) | 5.89  (0.10, 23.41) | 25.82  (6.75, 59.03) | 68.29  (31.49, 92.78) | |
| **Difference between simulated and baseline probabilities (%)** | | | | | |  |  | |
| *Changes in reimbursement rates* | | |  |  |  |  |  | |
|  | Tier 1: 5% increase | 1.01  (0.06, 3.61) | -0.44  (-2.13, -0.00) | -0.57  (-1.87, -0.05) | 0.72  (0.01, 2.57) | -0.24  (-1.17, 0) | -0.48  (-1.59, -0.01) | |
|  | Tier 2: 5% increase | -0.49  (-2.36, -0.01) | 4.01  (1.19, 6.72) | -3.52  (-5.96, -1.15) | -0.25  (-1.22, 0) | 2.53  (0.78, 4.79) | -2.28  (-4.36, -0.75) | |
|  | Tier 3: 5% increase | -0.49  (-1.61, -0.04) | -2.64  (-4.72, -0.78) | 3.14  (0.90, 5.53) | -0.41  (-1.27, -0.01) | -1.79  (-3.55, -0.54) | 2.20  (0.61, 4.15) | |
|  | Tier 3: 5% decrease | 0.51  (0.05, 1.62) | 2.75  (0.87, 4.74) | -3.25  (-5.59, -1.02) | 0.42  (0.01, 1.30) | 1.86  (0.57, 3.58) | -2.28  (-4.25, -0.65) | |
| *Changes in deductibles* | | |  |  |  |  |  | |
|  | Tier 3: 5% increase | 0.15  (0.00, 0.52) | 0.82  (0.00, 1.48) | -0.97  (-1.76, 0.00) | 0.11  (0.00, 0.38) | 0.49  (0.00, 0.94) | -0.60  (-1.17, 0.00) | |
|  | Tier 3: 50% increase | 1.52  (0.00, 4.99) | 8.51  (0.00, 14.94) | -10.02  (-17.45, 0.00) | 1.16  (0.00, 3.83) | 5.16  (0.00, 9.77) | -6.31  (-12.09, 0.00) | |
| *Changes in reimbursement rates (RR) and deductibles (DD)* | | | |  |  |  |  | |
|  | Tier 3: RR 5% decrease and DD 5% increase | 0.66  (0.06, 2.06) | 3.59  (1.07, 5.81) | -4.25  (-6.93, -1.29) | 0.54  (0.01, 1.60) | 2.37  (0.78, 4.25) | -2.91  (-5.09, -0.91) | |
| Simulated probabilities were only computed for significant effects of the reimbursement rates and deductibles on choice of hospital tier (c.f. **Figure 1**). Confidence intervals were estimated using 10 000 runs of the prediction on sets of random draws of model coefficients, and then taking the 2.5th and 97.5th percentiles for the 95% confidence interval. | | | | | | | | |

| **eTable 4: Relevance of patient characteristics and reimbursement ceiling (Relative rate ratios [95% CI]) to hospital tier choice for stroke admissions** | | | | | |
| --- | --- | --- | --- | --- | --- |
|  |  | **Urban areas** | | **Rural areas** | |
|  |  | **Tier 1 (ref: tier 3)** | **Tier 2 (ref: tier 3)** | **Tier 1 (ref: tier 3)** | **Tier 2 (ref: tier 3)** |
| Age group (ref: <55 years old) | |  |  |  |  |
|  | 55-64 years old | 1.20 (0.96, 1.50) | 1.26 (1.11, 1.42) | 0.94 (0.83, 1.06) | 0.91 (0.82, 1.02) |
|  | 65-74 years old | 1.35 (1.07, 1.71) | 1.38 (1.22, 1.56) | 1.02 (0.90, 1.16) | 1.02 (0.91, 1.15) |
|  | ≥75 years old | 1.30 (1.00, 1.69) | 1.33 (1.15, 1.52) | 1.30 (1.11, 1.53) | 1.19 (1.03, 1.37) |
|  | p-value for trend | 0.045 | <0.001 | <0.001 | 0.002 |
| Female | | 0.93 (0.78, 1.11) | 0.85 (0.77, 0.93) | 0.93 (0.83, 1.04) | 1.04 (0.94, 1.15) |
| Not married | | 1.07 (0.89, 1.28) | 1.02 (0.92, 1.13) | 1.18 (1.04, 1.33) | 0.96 (0.86, 1.07) |
| Household size | | 0.96 (0.91, 1.02) | 1.03 (1.01, 1.06) | 1.03 (1.00, 1.05) | 1.01 (0.98, 1.03) |
| Education level (ref: primary/middle school) | | |  |  |  |
|  | No formal school | 1.22 (1.00, 1.49) | 1.06 (0.94, 1.19) | 1.06 (0.96, 1.18) | 1.09 (0.99, 1.19) |
|  | High School or above | 1.05 (0.89, 1.23) | 0.89 (0.82, 0.97) | 0.80 (0.69, 0.93) | 0.80 (0.69, 0.92) |
|  | p-value for trend | 0.359 | 0.006 | 0.007 | <0.001 |
| Annual household income (ref: 10 000-19 999¥) | | |  |  |  |
|  | <10 000¥ | 1.04 (0.86, 1.26) | 1.20 (1.08, 1.34) | 1.02 (0.92, 1.13) | 1.02 (0.92, 1.11) |
|  | 20 000-34 999¥ | 0.90 (0.76, 1.06) | 0.95 (0.87, 1.04) | 0.82 (0.72, 0.94) | 0.91 (0.81, 1.02) |
|  | ≥35 000¥ | 0.72 (0.59, 0.88) | 0.80 (0.72, 0.89) | 0.70 (0.58, 0.84) | 0.69 (0.60, 0.80) |
|  | p-value for trend | 0.002 | <0.001 | <0.001 | <0.001 |
| Prior disease | |  |  |  |  |
|  | Ischaemic heart disease | 1.22 (1.03, 1.45) | 1.05 (0.95, 1.15) | 1.04 (0.92, 1.19) | 1.04 (0.92, 1.16) |
|  | Cerebrovascular disease | 1.32 (1.07, 1.62) | 1.07 (0.94, 1.21) | 1.08 (0.91, 1.27) | 1.10 (0.95, 1.27) |
|  | Cancer | 0.75 (0.54, 1.05) | 0.81 (0.67, 0.97) | 0.94 (0.73, 1.21) | 1.01 (0.81, 1.25) |
|  | Respiratory disease | 1.72 (1.44, 2.05) | 0.99 (0.88, 1.11) | 1.28 (1.13, 1.44) | 1.00 (0.90, 1.11) |
|  | Infectious and parasitic disease | 1.10 (0.74, 1.63) | 1.14 (0.91, 1.42) | 1.20 (0.97, 1.49) | 0.98 (0.81, 1.18) |
|  | Diabetes mellitus | 0.86 (0.73, 1.03) | 0.90 (0.83, 0.99) | 0.90 (0.79, 1.02) | 1.06 (0.95, 1.18) |
|  | Chronic kidney disease | 0.27 (0.07, 1.15) | 0.69 (0.43, 1.11) | 0.43 (0.22, 0.82) | 0.31 (0.18, 0.52) |
|  | Tuberculosis | 0.41 (0.12, 1.43) | 1.08 (0.67, 1.74) | 0.63 (0.34, 1.15) | 0.86 (0.53, 1.39) |
| Disease type (non-lacunar ischaemic stroke) | | |  |  |  |
|  | Lacunar stroke | 4.20 (3.55, 4.96) | 2.02 (1.84, 2.21) | 1.64 (1.40, 1.93) | 1.65 (1.43, 1.89) |
|  | Haemorrhagic stroke | 0.18 (0.12, 0.26) | 0.54 (0.47, 0.61) | 0.50 (0.44, 0.55) | 0.68 (0.62, 0.74) |
| Smoker | | 0.83 (0.69, 0.99) | 0.76 (0.69, 0.84) | 1.04 (0.93, 1.17) | 1.10 (0.99, 1.21) |
| Current alcohol drinker | | 0.91 (0.76, 1.09) | 0.97 (0.88, 1.07) | 0.92 (0.82, 1.03) | 0.99 (0.90, 1.10) |
| Overweight or obese | | 0.83 (0.73, 0.94) | 0.92 (0.86, 0.98) | 0.92 (0.85, 1.00) | 0.98 (0.91, 1.05) |
| Physical activity (MET/h) | | 1.01 (1.00, 1.02) | 1.00 (1.00, 1.01) | 1.00 (1.00, 1.00) | 1.00 (1.00, 1.00) |
| Self-rated health (ref: fair) | |  |  |  |  |
|  | Excellent | 1.13 (0.96, 1.33) | 0.96 (0.88, 1.05) | 0.86 (0.76, 0.98) | 0.89 (0.80, 1.00) |
|  | Good | 1.24 (1.05, 1.46) | 0.96 (0.88, 1.05) | 0.96 (0.87, 1.05) | 1.07 (0.98, 1.16) |
|  | Poor | 1.16 (0.93, 1.44) | 1.08 (0.96, 1.21) | 1.02 (0.91, 1.15) | 1.01 (0.90, 1.12) |
|  | p-value for trend | 0.296 | 0.085 | 0.019 | 0.467 |
| Log of reimbursement ceiling* | | 0.96 (0.76, 1.21) | 0.77 (0.68, 0.86) | 1.25 (1.05, 1.49) | 1.32 (1.12, 1.55) |
| Region fixed effects | | x | x | x | x |
| Year fixed effects | | x | x | x | x |
| ASC | | 0 (0, 0.04) | 0.12 (0.03, 0.45) | 0.23 (0.03, 1.50) | 0.13 (0.02, 0.77) |
| Number of cases | | 20302 | | 21130 | |
| Log-likelihood (final) | | -14188.0 | | -19957.0 | |
| AIC | | 28555.5 | | 40094.5 | |
| BIC | | 29367.1 | | 40909.6 | |
| ASC: tier-specific constant. AIC: Akaike information criterion, BIC: Bayesian information criterion. Results are from conditional logit models with the following tier-specific variables: reimbursement rate and deductible for each hospital tier. Only case-specific relative rate ratios are presented and interpreted as parameters of a binary logit model against the base category “Tier 3 hospital”. *The corresponding relative rate ratios associated with a 10% higher ceiling are: urban areas: exp(log(0.77)*log(1.1))=0.995 in tier 2 and rural areas: exp(log(1.25)*log(1.1))=1.004 in tier 1 and exp(log(1.32)*log(1.1))=1.005 in tier 2. Tier-specific odds ratios from this regression are presented in **Figure 1**. | | | | | |

| **eTable 5: Effects (95% CI) of changes in reimbursement rates and deductibles on probabilities of hospital tier choice for stroke and IHD in rural areas** | | | | | | | |
| --- | --- | --- | --- | --- | --- | --- | --- |
|  |  | **Stroke** | | | **IHD** | | |
|  |  | **Tier 1** | **Tier 2** | **Tier 3** | **Tier 1** | **Tier 2** | **Tier 3** |
| **Baseline probabilities (%)** | | 29.92  (6.19, 70.00) | 40.65  (18.58, 74.36) | 29.42  (9.87, 62.34) | 47.29  (4.98, 77.75) | 25.98  (8.74, 52.65) | 26.72  (9.87, 62.07) |
| **Difference between simulated and baseline probabilities (%)** | | | | |  |  |  |
| *Changes in reimbursement rates* | | |  |  |  |  |  |
|  | Tier 3: 5% decrease | 0.32  (0.05, 0.76) | 0.52  (0.06, 1.25) | -0.84  (-1.66, -0.27) | 0.33  (0.06, 0.81) | 0.25  (0.03, 0.90) | -0.58  (-1.32, -0.16) |
|  | Tier 3: 5% increase | -0.33  (-0.78, -0.05) | -0.52  (-1.26, -0.07) | 0.85  (0.27, 1.67) | -0.34  (-0.83, -0.06) | -0.25  (-0.90, -0.03) | 0.59  (0.16, 1.33) |
|  | Tier 3: 15% increase | -0.99  (-2.42, -0.15) | -1.58  (-3.80, -0.20) | 2.58  (0.83, 5.03) | -1.03  (-2.53, -0.19) | -0.75  (-2.70, -0.08) | 1.78  (0.47, 4.01) |
| *Changes in deductibles* | |  |  |  |  |  |  |
|  | Tier 1: 5% decrease | 0.20  (0.05, 0.50) | -0.12  (-0.30, -0.02) | -0.08  (-0.22, -0.01) | 0.10  (-0.04, 0.33) | -0.05  (-0.18, 0.02) | -0.05  (-0.18, 0.02) |
|  | Tier 1: 20% decrease | 0.79  (0.19, 2.00) | -0.47  (-1.20, -0.07) | -0.32  (-0.90, -0.06) | 0.42  (-0.16, 1.32) | -0.21  (-0.73, 0.08) | -0.21  (-0.72, 0.08) |
|  | Tier 1: 50% decrease | 2.02  (0.47, 5.04) | -1.20  (-3.02, -0.17) | -0.82  (-2.28, -0.15) | 1.05  (-0.41, 3.30) | -0.52  (-1.83, 0.20) | -0.53  (-1.81, 0.20) |
|  | Tier 1: No deductible | 4.15  (0.95, 10.35) | -2.46  (-6.21, -0.36) | -1.60  (-4.74, -0.31) | 2.79  (-0.81, 6.63) | -1.05  (-3.68, 0.39) | -1.06  (-3.64, 0.40) |
| *Changes in reimbursement rates (RR) and deductibles (DD)* | | | | |  |  |  |
|  | RR: Tier 3: +15% and  DD: Tier 1: -20% | -0.21  (-1.46, 0.94) | -2.02  (-4.12, -0.57) | 2.24  (0.59, 4.68) | -0.61  (-2.23, -0.64) | -0.95  (-2.86, -0.16) | 1.56  (0.20, 3.81) |
| Simulated probabilities were only computed for significant effects of the reimbursement rates and deductibles on choice of hospital tier (c.f. **Figure 1**). Confidence intervals were estimated using 10 000 runs of the prediction on sets of random draws of model coefficients, and then taking the 2.5th and 97.5th percentiles for the 95% confidence interval. | | | | | | | |

| **eTable 6: Relevance of patient characteristics and reimbursement ceiling (Relative rate ratios [95% CI]) to hospital tier choice for IHD admissions** | | | | | |
| --- | --- | --- | --- | --- | --- |
|  |  | **Urban areas** | | **Rural areas** | |
|  |  | **Tier 1 (ref: tier 3)** | **Tier 2 (ref: tier 3)** | **Tier 1 (ref: tier 3)** | **Tier 2 (ref: tier 3)** |
| Age group (ref: <55 years old) | |  |  |  |  |
|  | 55-64 years old | 0.97 (0.79, 1.19) | 1.02 (0.91, 1.15) | 0.85 (0.75, 0.96) | 0.98 (0.85, 1.13) |
|  | 65-74 years old | 0.96 (0.77, 1.20) | 1.05 (0.93, 1.19) | 0.90 (0.79, 1.02) | 1.11 (0.96, 1.28) |
|  | ≥75 years old | 0.91 (0.71, 1.17) | 1.02 (0.89, 1.18) | 0.99 (0.85, 1.15) | 1.27 (1.07, 1.50) |
|  | p-value for trend | 0.488 | 0.669 | 0.802 | 0.001 |
| Female | | 1.42 (1.17, 1.72) | 1.18 (1.07, 1.30) | 1.18 (1.07, 1.30) | 1.07 (0.94, 1.21) |
| Not married | | 0.94 (0.78, 1.14) | 0.98 (0.88, 1.09) | 1.17 (1.03, 1.32) | 1.08 (0.95, 1.23) |
| Household size | | 1.07 (1.00, 1.13) | 1.02 (0.99, 1.05) | 1.03 (1.00, 1.06) | 1.01 (0.98, 1.04) |
| Education level (ref: primary/middle school) | | |  |  |  |
|  | No formal school | 1.13 (0.90, 1.41) | 1.08 (0.95, 1.23) | 1.15 (1.03, 1.27) | 1.21 (1.08, 1.35) |
|  | High School or above | 0.99 (0.85, 1.17) | 0.90 (0.83, 0.98) | 0.72 (0.62, 0.84) | 0.81 (0.69, 0.96) |
|  | p-value for trend | 0.467 | 0.009 | <0.001 | <0.001 |
| Annual household income (ref: 10 000-19 999¥) | | |  |  |  |
|  | <10 000¥ | 1.40 (1.15, 1.70) | 1.09 (0.98, 1.23) | 1.08 (0.97, 1.20) | 1.09 (0.97, 1.22) |
|  | 20 000-34 999¥ | 0.86 (0.73, 1.01) | 0.89 (0.82, 0.98) | 0.92 (0.81, 1.04) | 0.99 (0.87, 1.13) |
|  | ≥35 000¥ | 0.61 (0.50, 0.75) | 0.81 (0.72, 0.90) | 0.57 (0.48, 0.68) | 0.70 (0.60, 0.82) |
|  | p-value for trend | <0.001 | <0.001 | <0.001 | <0.001 |
| Prior disease | |  |  |  |  |
|  | Cerebrovascular disease | 1.36 (1.19, 1.57) | 1.52 (1.40, 1.64) | 0.98 (0.89, 1.07) | 1.80 (1.64, 1.98) |
|  | Cancer | 0.63 (0.43, 0.93) | 0.71 (0.59, 0.86) | 1.08 (0.87, 1.33) | 1.06 (0.84, 1.32) |
|  | Respiratory diseases | 1.46 (1.22, 1.76) | 1.08 (0.96, 1.21) | 1.82 (1.64, 2.01) | 1.26 (1.13, 1.41) |
|  | Infectious and parasitic diseases | 0.87 (0.55, 1.39) | 1.31 (1.05, 1.63) | 1.32 (1.10, 1.57) | 0.93 (0.76, 1.14) |
|  | Diabetes mellitus | 0.74 (0.61, 0.89) | 0.93 (0.85, 1.02) | 0.96 (0.85, 1.08) | 1.19 (1.05, 1.35) |
|  | Chronic kidney disease | 0.12 (0.02, 0.83) | 0.91 (0.62, 1.33) | 0.39 (0.27, 0.57) | 0.41 (0.27, 0.63) |
|  | Tuberculosis | 0.36 (0.08, 1.58) | 0.79 (0.48, 1.31) | 0.74 (0.46, 1.20) | 1.08 (0.64, 1.83) |
| Other IHD (ref: AMI or angina) | | 1.70 (1.28, 2.25) | 1.34 (1.17, 1.53) | 1.85 (1.62, 2.11) | 1.39 (1.21, 1.59) |
| Smoker | | 0.83 (0.68, 1.01) | 0.84 (0.75, 0.93) | 0.89 (0.79, 0.99) | 0.90 (0.79, 1.01) |
| Current alcohol drinker | | 1.07 (0.88, 1.29) | 0.99 (0.89, 1.09) | 0.92 (0.82, 1.03) | 1.03 (0.91, 1.16) |
| Overweight or obese | | 0.89 (0.78, 1.01) | 0.94 (0.88, 1.01) | 0.79 (0.72, 0.86) | 0.88 (0.80, 0.97) |
| Physical activity (MET/h) | | 1.00 (0.99, 1.01) | 1.00 (1.00, 1.01) | 1.00 (1.00, 1.00) | 1.00 (0.99, 1.00) |
| Self-rated health (ref: fair) | |  |  |  |  |
|  | Excellent | 1.03 (0.88, 1.22) | 1.06 (0.97, 1.17) | 0.93 (0.81, 1.07) | 0.85 (0.73, 0.99) |
|  | Good | 0.99 (0.83, 1.17) | 1.02 (0.93, 1.12) | 1.05 (0.95, 1.16) | 1.18 (1.06, 1.30) |
|  | Poor | 0.88 (0.71, 1.10) | 1.04 (0.92, 1.17) | 1.01 (0.90, 1.13) | 1.06 (0.93, 1.20) |
|  | p-value for trend | 0.306 | 0.445 | 0.694 | 0.582 |
| Log of reimbursement ceiling | | 0.79 (0.62, 1.00) | 0.97 (0.86, 1.10) | 1.09 (0.92, 1.30) | 0.86 (0.71, 1.04) |
| Region fixed effects | | x | x | x | x |
| Year fixed effects | | x | x | x | x |
| ASC | | 0.06 (0, 1.01) | 0.03 (0.01, 0.11) | 6.48 (0.89, 47.19) | 7.55 (0.94, 60.48) |
| Number of cases | | 19283 | | 17890 | |
| Log-likelihood (final) | | -13241.90 | | -16863.30 | |
| AIC | | 26655.77 | | 33898.57 | |
| BIC | | 27426.81 | | 34663.16 | |
| ASC: tier-specific constant. AIC: Akaike information criterion, BIC: Bayesian information criterion. Results are from conditional logit models with the following tier-specific variables: reimbursement rate and deductible for each hospital tier. Only case-specific relative rate ratios are presented and interpreted as parameters of a binary logit model against the base category “Tier 3 hospital”. Tier-specific odds ratios from this regression are presented in **Figure 1**. | | | | | |

| **eTable 7: Associations (Odds Ratios [95% CI]) between out-of-pocket (OOP) payments or actual reimbursement rates (ARR) and choice of hospital tier for admissions for stroke and IHD** | | | | | | | | |
| --- | --- | --- | --- | --- | --- | --- | --- | --- |
|  |  | **Stroke** | | | | **IHD** | | |
|  |  | **OOP payments**  **(per 100¥)** | | **ARR**  **(per % point)** | | **OOP payments**  **(per 100¥)** | | **ARR**  **(per % point)** |
| **Urban areas** | | |  | |  | |  | |
|  | **Tier 1** | 0.99 (0.97, 1.01) | | 1.01 (0.99, 1.02) | | 0.99 (0.97, 1.00) | | 1.00 (0.98, 1.02) |
|  | **Tier 2** | 0.98 (0.97, 0.99) | | 1.02 (1.01, 1.03) | | 0.99 (0.98, 1.00) | | 1.01 (0.99, 1.02) |
|  | **Tier 3** | 0.98 (0.98, 0.99) | | 1.02 (1.01, 1.03) | | 1.00 (0.99, 1.00) | | 1.00 (0.99, 1.02) |
| **Rural areas** | | |  | |  | |  | |
|  | **Tier 1** | 1.00 (0.99, 1.02) | | 1.01 (1.00, 1.02) | | 1.01 (1.00, 1.03) | | 0.99 (0.98, 1.01) |
|  | **Tier 2** | 1.00 (0.99, 1.00) | | 1.00 (1.00, 1.01) | | 0.99 (0.99, 1.00) | | 1.02 (1.01, 1.03) |
|  | **Tier 3** | 0.99 (0.98, 0.99) | | 1.02 (1.02, 1.03) | | 0.99 (0.99, 0.99) | | 1.02 (1.02, 1.03) |
| Models for out-of-pocket payments and actual reimbursement rates are different regressions. Results are from conditional logit models with the following tier-specific variables: interactions between tier and out-of-pocket payments or actual reimbursement rates, and as case-specific variables: demographic, socioeconomic, lifestyle and morbidity factors and log reimbursement ceiling. Only tier-specific odds ratios are presented and interpreted as follows: an odds ratio > 1 means that if the regressor increases for one hospital tier, then that hospital tier is chosen more; and vice versa for an odds ratio < 1. | | | | | | | | |

**References**

1. Train K. Discrete Choice Methods With Simulation. 2nd ed: Cambridge University Press; 2009.
2. Train KE. Qualitative Choice Analysis: Theory Econometrics, and an Application to Automobile Demand: MIT Press; 1986.
3. Cheng S, Long JS. Testing for IIA in the Multinomial Logit Model. *Sociological Methods & Research*. 2007; **35**(4): 583-600.
4. National Bureau of Statistics of China. China Statistical Yearbook. 2018 [cited 2021 27 July 2021]; Available from: <http://www.stats.gov.cn/english/Statisticaldata/AnnualData>
